# Proteome-wide Mendelian randomisation of lung function to identify potential therapeutic targets for respiratory disease

**DOI:** 10.1183/23120541.00828-2025

**Published:** 2026-09-01

**Authors:** Jing Chen, Nick Shrine, Kayesha Coley, Richard J. Packer, Ahmed Edris, Abril G. Izquierdo, Brandon Lim, Mikyeong Lee, Frank Dudbridge, Robin G. Walters, Ian P. Hall, Louise V. Wain, Martin D. Tobin, Anna L. Guyatt, the SpiroMeta Consortium and the CHARGE consortium

**Affiliations:** 1 Division of Public Health and Epidemiology, School of Medical Sciences, University of Leicester, Leicester, UK; 2 National Institute for Health Research, Leicester Respiratory Biomedical Research Centre, University of Leicester, Leicester, UK; 3 Nuffield Department of Population Health, University of Oxford, Oxford, UK; 4 Epidemiology Branch, National Institute of Environmental Health Sciences, National Institutes of Health, Research Triangle Park, NC, USA; 5 MRC Population Health Research Unit, University of Oxford, Oxford, UK; 6 Division of Respiratory Medicine and NIHR Nottingham Biomedical Research Centre, University of Nottingham, Nottingham, UK

## Abstract

**Background:**

Despite multiple clinical trials, disease-modifying treatments for COPD are currently limited. Since many drugs target proteins, identifying causality between proteins and lung function informs understanding of COPD pathophysiology and may suggest novel targets. We used Mendelian randomisation (MR) to prioritise proteins as potentially causal for imparied lung function. For prioritised proteins, we explored their potential suitability as drug targets by predicting their effects on a range of clinical outcomes.

**Methods:**

We used genome-wide association study (GWAS) data on 2923 proteins (n=48 195, UK Biobank) to identify single genetic variants (protein quantitative trait loci (cis-pQTLs)) associated with protein levels (p≤5×10^−9^, variant ≤100 kb of a transcription start site). We performed cis-pQTL-MR analyses of four spirometric traits (n=149 166, 36 independent cohorts). Sensitivity analyses included colocalisation and reverse direction MR. We report associations between cis-pQTLs for prioritised proteins and multiple clinical respiratory outcomes, and use phenome-wide analysis to explore potential adverse effects or drug repurposing opportunities.

**Findings:**

1841 proteins had a suitable cis-pQTL. We implicated 16 proteins as potentially causal for lung function (p<1.71×10^−5^): seven proteins have not been implicated by previous lung function GWAS or MR (CCND2, DTD1, PILRA, PTPRK, TDRKH, GRHPR, NUDT5), and we provide corroborative evidence for 10 proteins. We add to the literature identifying surfactant protein D (SFTPD) as a candidate, yet predict that integrin subunit alpha V (ITGAV) inhibition could impair some lung function measures, mimicking adverse results from a recent trial.

**Interpretation:**

Our approach identifies proteins (some novel) that are potentially therapeutic targets for respiratory disease, and which warrant follow-up for utility and safety.

## Shareable abstract

**Two-sample Mendelian randomisation of protein levels and spirometry were used to infer potential causal drivers of respiratory disease. These include some novel proteins that may warrant follow-up for utility and safety as drug targets.** https://bit.ly/4dbZ9bY

## Introduction

Impaired lung function predicts mortality and is a diagnostic criterion for COPD, the sixth leading cause of global disability-adjusted life-years [1]. Although β_2_ adrenoreceptor agonists, antimuscarinic agents and corticosteroids are currently successfully used in the treatment of COPD, there has been a resurgence in drug development for COPD, with multiple clinical trials in progress for biologics and the first Food and Drug Administration (FDA)-approved biologic, dupilumab [2]. One approach to identifying drug targets involves the use of genetic association studies, which reported 1020 independent associations with lung function and COPD [3–5]. Protein associations with lung function may identify drug targets, as many pharmaceuticals act on proteins. Indeed, a proteomic-based score has been shown to predict COPD onset [6]. However, confounding and reverse causation typically affect such observational studies, which can result in noncausal protein–lung function associations being prioritised and causal lung function associations being overlooked.

Integration of genomic and proteomic data enables inference of circulating protein associations with lung function [7, 8]. Genetic variants that act on levels of a specific protein (protein quantitative trait loci (pQTLs)) are often located near the gene encoding that protein [9]. These cis-pQTLs are often strongly associated with a protein level, with a direct and clear mechanism, making them suitable as instruments for Mendelian randomisation (MR) to inform drug target identification [7].

We aimed to integrate data on genomic variation and proteins in 50 395 UKB participants with data on genomic variation and lung function in 149 166 participants from 36 cohorts to estimate the predicted effect of protein levels on four lung function traits, *via* strictly defined cis-pQTLs. Whilst the proteomics of lung function has been studied in smaller sample sizes [10] and limited numbers of traits [11], our study draws upon the largest genome-wide association studies (GWAS) of lung function. We discuss what is known about how the identified proteins may influence the pathophysiology of obstructive lung disease and their potential utility when considering these proteins as drug targets.

## Methods

### Genetic association analyses for proteomics

The UK Biobank Pharma Proteomics Project (UKB-PPP) characterised the plasma proteomic profiles of 54 306 UKB participants. Sample processing and proteomic measurement quality control in UKB-PPP is detailed elsewhere [12]. We undertook ancestry-specific GWAS of 2923 proteins in 50 395 participants, including 48 195 European (EUR) participants, plus 1202 African (AFR), 482 American (AMR), 237 East Asian (EAS) and 729 South Asian (SAS) ancestry participants in UK Biobank (ancestry described in Shrine *et al.* [5]). Relative protein abundancies were log_2_-transformed, then adjusted using linear regression for age, sex and protein assay batch. GWAS were performed on untransformed protein residuals using REGENIE v3.2.6 *via* a two-step procedure to account for population structure [13], including covariates of UKB genetic array and the first 10 genetic principal components. For MR analyses, effect sizes of single nucleotide polymorphisms (SNPs) on proteins were converted to changes in standard deviations.

### Single-cis-MR analysis

**FIGURE 1:**
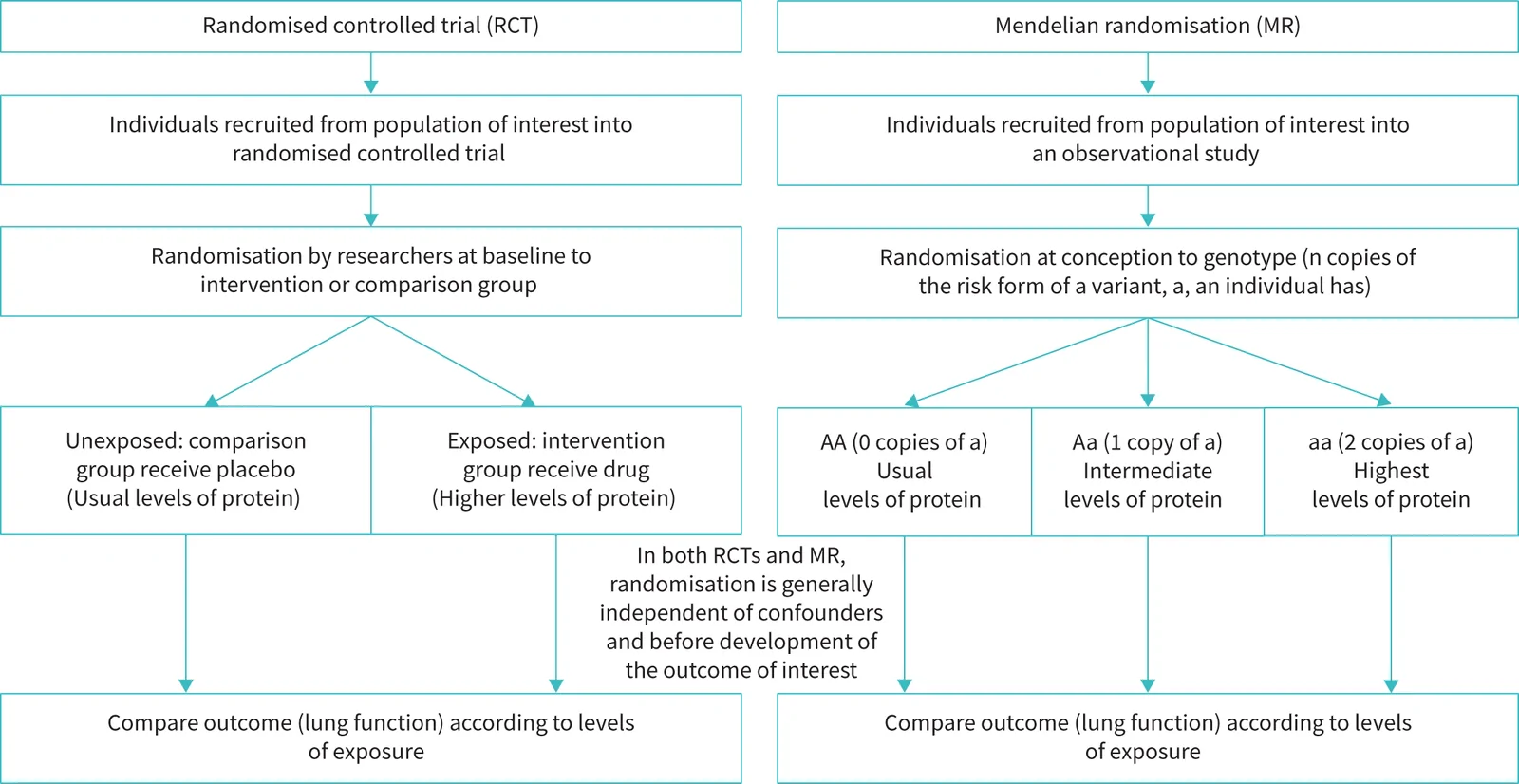
Diagram demonstrating the analogy of Mendelian randomisation (MR) using a single genetic variant predicting protein levels, to a randomised controlled trial (RCT) of a drug intervention to alter protein levels. Both RCTs and MR studies are forms of the causal inference technique, instrumental variable analysis. In an RCT, individuals are recruited from a sample of interest into an interventional trial, whereas MR studies often taken place in the context of an existing population-based cohort study. In an RCT, researchers use randomisation to assign individuals to receive an intervention (for example, a drug that alters levels of a given protein), or to a comparison, which may be a placebo, or gold-standard treatment (the usual levels of protein). In MR, “nature's randomisation” is at the moment of conception where individuals are assigned to “genotypes”, *e.g.* at a given position in the genome, individuals may have 0 (AA), 1 (Aa), or 2 (aa) copies of one form of a genetic variant (allele), “a” – often the rarer form in a population being designated as “a”. In the example, and for the ease of explaining the analogy, here both “a” and the drug increase levels of a protein, but this need not necessarily be the case. Importantly, MR uses genetic variation to mimic the effect of intervening to modify protein levels in a trial setting. In both examples, randomisation (to intervention/comparison in the RCT setting, or genotype in the MR setting) is generally before development of the outcome of interest, and independent of confounders (in the MR setting, this is because of random allocation at conception and Mendel's law of independent assortment, respectively). In both settings, outcomes are then compared by levels of exposure.

**FIGURE 2:**
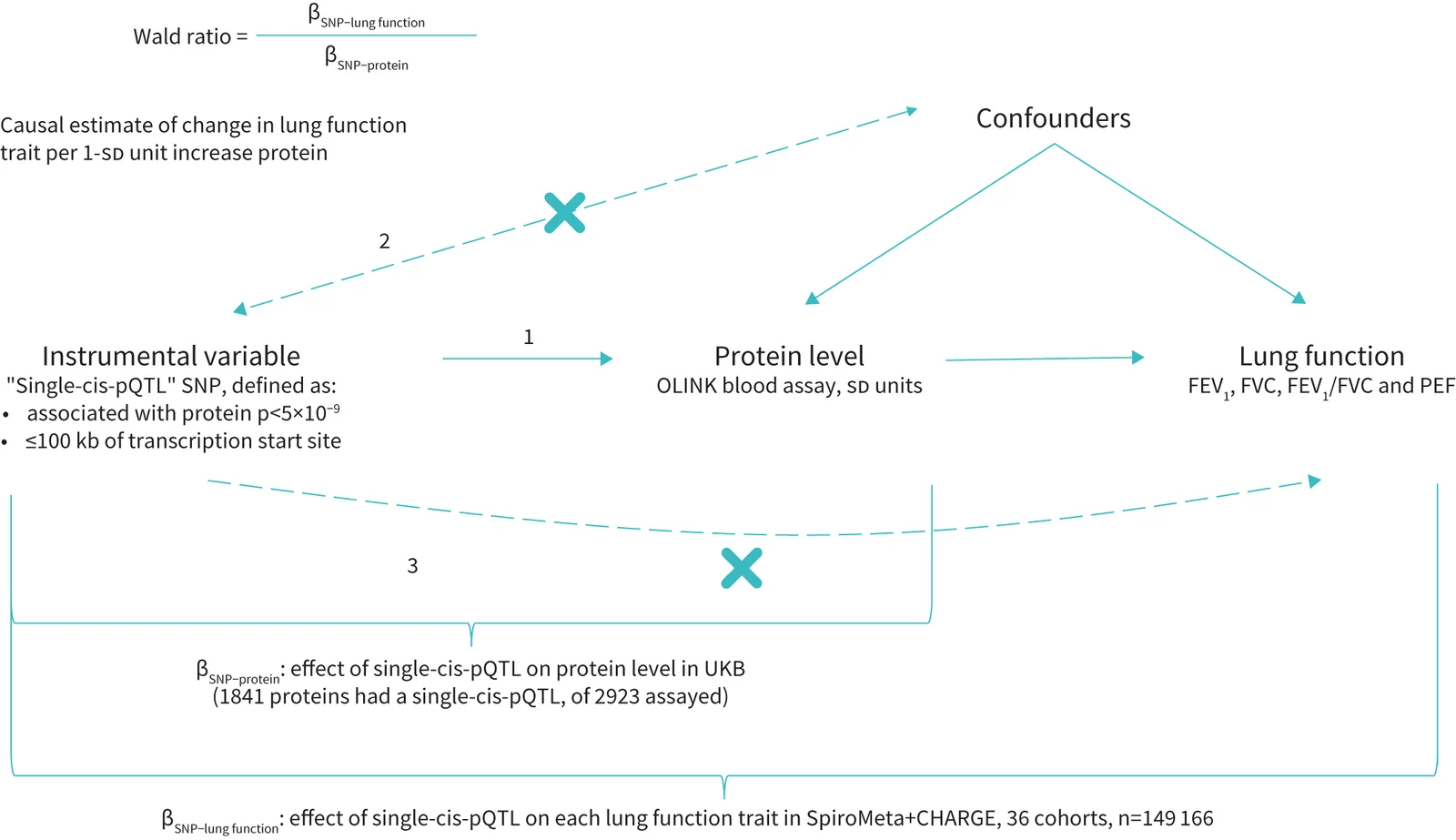
We performed two-sample Mendelian randomisation (MR) analyses to estimate causal effects of 1841 proteins on four lung function traits: forced expiratory volume in 1 s (FEV_1_), forced vital capacity (FVC), their ratio (FEV_1_/FVC) and the peak expiratory flow (PEF). The assumptions of MR are shown in the directed acyclic graph. MR is able to identify causal associations if: 1) the instrumental variable are associated with the exposure of interest (here, protein levels); 2) the instrumental variable is not associated with unobserved confounders of the exposure–outcome association (straight dashed arrow) and 3) the only association between the genetic variants and the outcome is *via* the exposure, and not *via* an alternate pathway (*i.e.* no “horizontal pleiotropy”, curved dashed arrow). As it is difficult to verify this last assumption, we focused this study on protein quantitative trait loci (cis-pQTLs) which are more likely to be valid instruments [7], since these variants are less likely to violate this assumption than trans-pQTLs. Strictly defined single-cis-pQTL single-nucleotide polymorphisms (SNPs) were sought for use as instrumental variables (IVs) for 2923 proteins (1841 proteins had a valid IV). Causal estimates, as change in the lung function trait per 1-sd protein increase, were calculated for each protein–lung function trait pair using a Wald ratio estimator, by dividing the effect on the IV on lung function (estimated in the SpiroMeta+CHARGE consortia, n=149 166) by the effect of the IV on protein level (estimated in UK Biobank (UKB) EUR participants, n=48 195).

The principle of MR is explained in figure 1, using the analogy of a randomised controlled trial (figure 2). We selected strictly defined cis-pQTL instruments, defined as SNPs most strongly associated (p≤5×10^−9^) with a protein level within 100 kb of a protein's cognate gene transcription starting site. Using the Two-Sample MR package in R, we applied two-sample MR by integrating the protein (exposure) EUR genetic association data from UKB with lung function trait (outcome) GWAS data (forced expiratory volume in 1 s (FEV_1_), forced vital capacity (FVC), FEV_1_/FVC and peak expiratory flow (PEF)) from meta-analyses of 36 non-UKB cohorts of 149 166 European participants [5]. We implemented single-cis-MR analysis which utilised the strongest cis-pQTL instrument, and the Wald ratio [14] to estimate the causal effect of each protein on lung function. We prioritised for follow-up protein–lung function associations exceeding p<1.71×10^−5^ (Bonferroni correction for 2923 proteins). We do not report proteins encoded by genes in the human major histocompatibility complex (chromosome 6, 26–34 Mb) due to extensive linkage disequilibrium (LD). We report all coordinates using GRCh37/hg19. Methods for colocalisation, reverse causation, respiratory-wide association studies and PheWAS are detailed in the supplementary Methods.

### Replication analysis

Proteins identified from the single-cis-MR analysis were further investigated in a replication analysis using the SomaLogic-derived pQTL data from deCODE [15] and INTERVAL [16] studies. We applied the same two-sample MR approach by integrating pQTL data for proteins available from either deCODE or INTERVAL with the lung function trait GWAS data from UK Biobank European population. Protein–lung function associations that met a Bonferroni-corrected significance threshold for the number of proteins prioritised from the MR were considered replicated.

### Druggability

We queried the gene–drug interaction table from the Drug–Gene Interaction Database for each prioritised protein; associated drugs were linked to ChEMBL IDs, and indications annotated using MeSH headings.

### Associations of top proteins in diverse ancestries

For proteins prioritised by the single cis-pQTL MR analyses in EUR participants, we explored whether the same cis-pQTL was associated at p<0.05, within ancestry subgroups defined in Shrine *et al.* [5].

## Results

**FIGURE 3:**
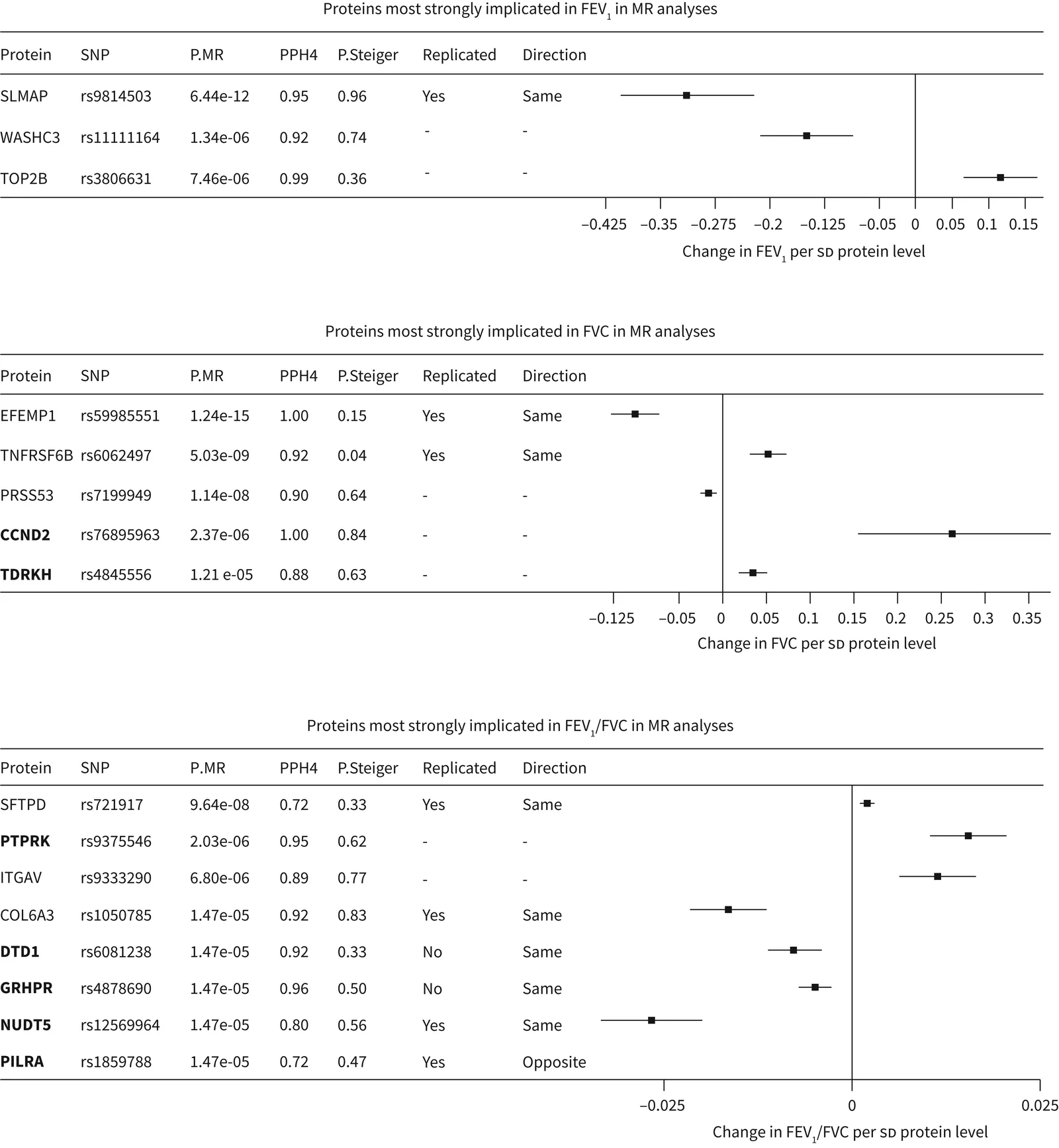
The 16 proteins implicated by the single-cis-MR analyses for the lung function traits. Forest plots of the Wald ratio estimates for the strongest associated lung function trait are shown, along with the p-value for the Wald ratio method (P.MR), the posterior probabilities of colocalisation (PPH4), the p-value for the reverse causation test (P.Steiger) and replication results (Replicated and Direction, with a “-” indicating that replication data were not available). Proteins which have not been previously implicated by a lung function signal are highlighted in bold. Boxes of the forest plot represent effect sizes (change in the lung function trait in native units: L for FEV_1_ and FVC and unitless ratio for FEV_1_/FVC), whiskers are 95% CIs. FEV_1_: forced expiratory volume in 1 s; FVC: forced vital capacity; MR: Mendelian randomisation; SNP: single-nucleotide polymorphisms; sd: standard deviation.

Our GWAS of 2923 proteins identified 1841 proteins with a suitable cis-pQTL instrument. The cis-pQTL MR analyses causally implicated 16 proteins in lung function (p<1.71×10^−5^), including seven proteins not implicated by previous GWAS, *i.e.* where the pQTL SNP was independent of reported lung function signals (CCND2, DTD1, PILRA/PILRB, PTPRK, TDRKH, GRHPR and NUDT5) (figure 3, supplementary Tables S3 and S4) [5]. All proteins were supported by strong genetic instruments (mean F-statistic=4915; minimum F-statistic=35; supplementary Table S3), suggesting negligible weak-instrument bias. Nine of the proteins identified were causally implicated in lung function by SNPs in LD with known lung function signals (COL6A3, EFEMP1, ITGAV, PRSS53, SFTPD, SLMAP, TNFRSF6B, TOP2B and WASHC3) [17]. Of the 16, eight proteins (CCND2, COL6A3, EFEMP1, ITGAV, SFTPD, SLMAP, TNFRSF6B and WASHC3) have also been reported as having evidence from MR, plus colocalisation [10, 11].

For 12 out of 16 proteins (CCND2, DTD1, EFEMP1, ITGAV, PILRA/B, PRSS53, PTPRK, SLMAP, TDKRH, TNFRSF6B, TOP2B and WASHC3), we found that the cis-pQTL showed association (p<0.01) with at least one of COPD, emphysema, asthma, idiopathic pulmonary fibrosis (IPF) onset or diffusing capacity of the lung for carbon monoxide (*D*_LCO_), interstitial lung abnormalities (ILA), or chronic sputum production (supplementary Tables S5 and S6). Although no associations with SFTPD were observed in the respiratory PheWAS, its pQTL was previously reported as associated with emphysema [17].

For FEV_1_ the strongest association was for the sarcolemma-associated protein, SLMAP. An increase in SLMAP predicted by rs9814503 was associated with reduced FEV_1_ (p=6.44×10^−12^), with weaker associations with reduced FEV_1_/FVC (p=6.33×10^−5^) and reduced FVC (p=1.05×10^−5^). The SLMAP-increasing allele (C) of rs9814503 was associated with increased risk of asthma, childhood-onset asthma and COPD (supplementary Tables S2, S5 and S6) in the respiratory PheWAS, with reduced diastolic blood pressure in the PheWAS (supplementary Table S7), and SNPs in this gene (including rs17666667-T, r^2^=0.99 with rs9814503-C) are associated with reduced smoking initiation [18]. We note that the C allele of rs9814503 is also associated with reduced FEV_1_, FVC, FEV_1_/FVC and PEF in never-smokers; therefore, the SNP–lung function association is not *via* smoking [5]. We previously showed a consistent FEV_1_ association in children (rs9872373-C (correlated with C allele of rs9814503, r^2^=0.98), −0.015, se=0.004, p=0.0006) [5]. This pattern of association with phenotypes at different stages of life could be consistent with a mechanism influencing airflow obstruction in early life, then tobacco avoidance and fixed airflow obstruction in later life.

Additional predicted protein–FEV_1_ associations were seen for WASHC3 (WASH Complex Subunit 3) and TOP2B (DNA Topoisomerase II β). pQTLs showed association with decreased sputum production for WASHC3 and reduced risk of COPD for TOP2B.

For FVC, we observed the strongest association with EFEMP1(EGF Containing Fibulin Extracellular Matrix Protein 1). An increase in EFEMP1 predicted by rs59985551 was associated with lower FVC (p=1.24×10^−15^) (see also Aggarwal *et al.* [10]), increased FEV_1_/FVC (p=1.52×10^−7^) and decreased emphysema risk. EFEMP1 is associated with elastic fibre formation, and in the PheWAS, consistent with reduced tissue elasticity, the EFEMP1-increasing allele rs59985551 (T) showed association with reduced height, reduced hernia risk, reduced diverticular disease and increased carpal tunnel syndrome.

TNFRSF6B is a member of the tumour necrosis factor receptor superfamily and acts as a decoy receptor to neutralise cytotoxic ligands. An increase in TNFRSF6B predicted by rs6062497 was associated with increased FVC (p=5.03×10^−9^) and FEV_1_ (p=2.26×10^−8^) (see also Aggarwal *et al.* [10]), and the protein-increasing allele (C) was associated with multiple respiratory traits, including reduced risk of asthma, COPD, IPF and bronchitis, and increased pneumothorax risk. Notably, rs6062497 is downstream of RTEL1 (telomere elongation helicase); telomere genes influence interstitial lung disease risk [19]. In PheWAS, the protein-increasing allele is associated with lower granulocytes (eosinophils and neutrophils), lower C-reactive protein, reduced smoking heaviness and smoking onset, reduced asthma risk and reduced inflammatory bowel disease risk. The same allele was associated with increased dental disorders and traits related to tissue laxity, such as diverticulosis.

For PRSS53 (serine protease 53), an increase in levels was instrumented by rs7199949, and associated with similar decreases in FVC (p=1.14×10^−8^) and FEV_1_ (p=1.46×10^−8^). The PRSS53-increasing allele was also associated with increased asthma risk, especially in children, and COPD. In PheWAS, the same allele was associated with many traits, including but not limited to those relating to increased body size, altered red cell traits (and increased HbA1C without association with diabetes), reduced diverticular disease, reduced protein levels (including albumin), reduced vitamin D, and lipids.

An increase in CCND2 (G1/S-specific cyclin-D2, fundamental to the cell cycle) predicted by rs76895963 was associated with higher FVC (p=2.37×10^−6^). In the respiratory PheWAS, the protein-increasing allele (G) was associated with increased risk of COPD and ILA and reduced *D*_LCO_ in IPF. PheWAS showed that the protein-increasing allele was also associated with growth-related traits, including increased height and weight, plus reduced type 2 diabetes risk.

TDRKH (Tudor And KH Domain Containing) was associated with increased FVC (p=1.21×10^−5^) and more weakly with increased FEV_1_ (p=0.00032), and the protein-increasing allele (A) was associated with reduced childhood-onset asthma (p=3.4×10^−9^) in the respiratory PheWAS and with reduced γ-glutamyltransferase in the broader PheWAS.

The protein-increasing allele (A) of a nonsynonymous (Met11Thr) SNP in SFTPD (rs721917, surfactant protein D) was associated with increased FEV_1_/FVC. SFTPD was previously implicated in MR analyses of COPD [10, 20], and rs721917 associations with emphysema have been reported [17]; we corroborate these results in a disease-relevant quantitative trait, and show that this is not driven by reverse causation. Our PheWAS suggests that this variant has a specific effect on lung function, with no nonrespiratory traits associated in the PheWAS at a false discovery rate (FDR) <1%. We found modest associations (0.01<p<0.05) of the protein-increasing allele (A) with decreased COPD, chronic bronchitis, emphysema, respiratory infections and asthma that support previous findings [20], and the same allele was reported as protective against severe COVID-19 [21, 22]. SFTPD is involved in innate immunity, surfactant regulation and lipid homeostasis [20]. We found consistent evidence of SNP–SFTPD associations between UK Biobank (Olink) and China Kadoorie Biobank (Olink and Somascan), but the same protein-increasing allele decreased FEV_1_/FVC in deCODE [15].

PTPRK (protein tyrosine phosphatase receptor type K) regulates cell contact and adhesion, and negatively regulates EGFR signalling. The protein-increasing allele was associated with improved FEV_1_/FVC and reduced asthma risk, but with increased abdominal aortic aneurysm risk, suggesting caution in considering PTPRK as a druggable target.

Our MR results for ITGAV (Integrin Subunit Alpha V) provide causal evidence counter to previous eQTL evidence [4, 5, 23] but consistent with recent trial evidence [24]. ITGAV is a druggable target (supplementary Table S8). The protein-increasing allele (rs9333290-G) was associated with increased FEV_1_/FVC and PEF, lower asthma, COPD and emphysema risk, reduced height, and increased phosphate.

The Collagen Type VI Alpha 3 Chain (COL6A3) is found in most connective tissues. We found that higher COL6A3 levels, as predicted by rs1050785-A, were causally associated with lower FEV_1_/FVC (p=1.47×10^−5^), but the same SNP was not associated with any clinical phenotype at p<0.01.

DTD1 (D-Aminoacyl-TRNA Deacylase 1) is involved in initiating DNA replication. The allele (G) associated with increased DTD1 was linked with lower FEV_1_/FVC, increased COPD risk, and raised monocytes and creatinine levels in the broader PheWAS. Higher GRHPR (Glyoxylate And Hydroxypyruvate Reductase) and NUDT5 (Nudix Hydrolase 5) levels were both associated with lower FEV_1_/FVC; however, neither of the SNPs that act as instruments for these proteins were associated (p<0.01) with any clinical respiratory trait. The NUDT5 cis-pQTL, rs12569964, was associated with height and Type 2 diabetes traits, but no PheWAS associations were observed for GRHPR at FDR<1%.

The cis-pQTL for paired immunoglobin like type 2 receptor alpha (PILRA) and beta (paralog PILRB), involved in immune system signal transduction and herpes simplex virus cellular entry, was a nonsynonymous SNP, rs1859788. Our MR analyses showed an association between increased PILRA and increased FEV_1_/FVC and similarly, an association between PILRB and increased FEV_1_/FVC. The PILRA/PILRB-increasing allele (G) was associated with lower risk of emphysema, COPD and asthma and notably increased chronic sputum production. However, in the broader PheWAS, the protein-increasing allele (G) was associated with an HbA1c increase; this allele has been reported as associated with an *increased* risk of dementia [25], which could caution against PILRA or PILRB as respiratory drug targets.

Of the 16 proteins we highlight, nine were available to assess replication in SomaLogic-derived proteomics resources (supplementary Table S9). For seven of these nine proteins (SLMAP, PILRA, COL6A3, EFEMP1, NUDT5, SFTPD and TNFRSF6B), MR-derived protein–lung function associations met our replication threshold (p<3.13×10^−3^, correcting for 16 proteins, supplementary Table S9). One of these proteins, NUDT5, replicated and has not been implicated by a previous lung function signal, although a study by Aggarwal *et al.* [10] did not find evidence of colocalisation (and thus was not reported as a main result). For PILRA (predicted by a nonsynonymous SNP), the MR-derived protein–lung function association in the SomaLogic-derived replication analyses had an opposite direction of effect (the protein-increasing allele reduced FEV_1_/FVC). For the remaining two proteins (DTD1 and GRHPR) that did not reach significance in the replication analysis, we observed a consistent effect direction.

For the 16 proteins we highlight (counting PILRA/PILRB as one protein), we explored whether the top cis-pQTL for each protein was strongly associated in four other ancestry subgroups in UK Biobank (AFR, AMR, EAS and SAS). For the AFR, AMR, EAS and SAS subgroups, respectively, the number of SNPs (out of the SNPs available for that subgroup, maximum n=16) significant for their respective protein level at p<0.05 was eight out of 16 for AFR (n=1185), nine out of 15 for AMR (n=479), two out of nine for EAS (n=235) and eight out of 15 for SAS (n=719) (supplementary Table S10). Of note, the cis-pQTL SNPs for PILRA/PILRB and SFTPD were available in all five subgroups, and were strongly associated with these protein levels in all subgroups. Importantly, we note that the SNPs for these proteins were missense SNPs, meaning that they may be more likely to be the causal variant driving protein levels.

## Discussion

We integrated genomic and proteomic data from UK Biobank to study the causal effect of proteins on lung function traits measured by spirometry. Instead of using observational epidemiological associations to prioritise protein–outcome pairs for study, we applied a hypothesis-free agnostic approach, identifying strictly defined cis-pQTLs for as many proteins as possible. We identified 16 proteins with evidence of a causal effect on lung function, of which seven had not been previously identified in large-scale lung function GWAS. Eight of the 16 proteins (CCND2, COL6A3, EFEMP1, ITGAV, SFTPD, SLMAP, TNFRSF6B and WASHC3) have been identified in other recent MR efforts with colocalisation [10, 11]. Our approach confirms the utility of a “genetics-first” approach for identifying protein–disease associations, and provides a shortlist of proteins worthy of exploration as therapeutic targets.

Results from eQTL and pQTL do not always tally [26], but our cis-pQTL results enable triangulation of evidence. Notably, the evidence about the effect of ITGAV on lung function from pQTL conflicts with that from eQTL. In a previous paper, increased *ITGAV* expression was associated with decreased lung function [4], yet subsequent trials reported that ITGAV inhibition led to pulmonary fibrosis exacerbations and lung function decline [24]. Our current MR results (see also Aggarwal *et al.* [10]) would have predicted the results of this trial.

We previously showed that studying lung function is a well-powered way of studying relevant chronic respiratory diseases, such as COPD and IPF [5, 27]. To explore the relevance of protein–lung function associations to clinical disease phenotypes, we performed a “respiratory-wide association study” (Respiratory PheWAS) and found that many of our cis-pQTL SNPs were associated with a clinically relevant outcome, such as asthma, COPD (and sub-components emphysema and bronchitis) and interstitial lung disease, and clinically relevant symptoms such as sputum production.

Whilst our results suggest a causal role for all of the proteins we prioritise in lung function and clinical end-points, their suitability as therapeutic targets varies. The proteins identified in both our analysis and prior studies [10, 20], such as ITGAV and SFTPD, have a known role in the lung, are expressed in pulmonary tissue and had few unwanted predicted effects in our PheWAS, strengthening evidence for these proteins as therapeutic targets. Other proteins, such as WASHC3 and TOP2B, have such fundamental roles that they seem implausible as therapeutic targets to improve lung function (topoisomerase inhibitors are chemotherapeutic agents with low therapeutic indices and wide-ranging effects). Finally, our PheWAS approaches provide corroborative insight into the biology of some of the putative targets suggested. For example, the PheWAS suggests that inhibiting EFEMP1 [10] could improve FVC and reduce risk of pulmonary fibrosis, but increase risk of traits related to tissue laxity. Thus, integrating PheWAS data is helpful for understanding wider consequences of modulating the protein target.

Strengths of our work include our strict cis-pQTL definition to mitigate against risk of horizontal pleiotropy (figure 2) and requirement for corroborating evidence from colocalisation [28]. Instruments for a protein of interest that are strong and have a highly plausible, specific role to play are useful in MR [7]. The UKB-PPP project measured a subset of the human proteome, and our strict threshold for association further limited the number of proteins with suitable instrumental variables. Since we hypothesised that any protein could be causal for lung function, our discovery Bonferroni correction threshold incorporated the number of proteins assayed, *i.e.* stricter than the number of proteins with cis-pQTLs. Others have advocated different discovery thresholds, *i.e.* p<0.005, for hypothesis-free analyses, yet the UKB-PPP measured proteins are not a random sample of the proteome and may have a higher prior probability of causality. We used a very large resource of proteomics data and the largest set of lung function GWAS results available and we avoided sample overlap, which can bias MR results. We also assessed for reverse causation. We used discovery SNP–protein results from a European subset, as sample sizes for other ancestries were small. We observed concordant associations across ancestry subgroups for some proteins proxied by missense SNPs. Yet, absence of concordant associations for other SNP–protein pairs across other ancestries may reflect some combination of SNP availability, statistical power, and whether or not the cis-pQTL instrumental variable is in LD with the causal SNP in non-EUR populations. Another strength is that we were able to explore corroborative evidence for over half of our prioritised proteins by using independent replication from other sources.

We acknowledge limitations. First, our rationale for single-cis-MR focus necessarily precludes multi-SNP MR, which may limit statistical power and will constrain evaluation of horizontal pleiotropy introduced by multi-SNP MR. We prioritised the strongest, strictly defined cis-pQTL for each protein to maximise instrument strength and minimise weak instrument and pleiotropic bias. Although multi-SNP MR methods can, in principle, account for correlated variants and pleiotropy, these approaches rely on additional modelling assumptions about the distribution of latent pleiotropic effects and were developed for polygenic exposures with genome-wide instruments. Their performance in cis-MR analysis is not established. While recent methodological advances tailored to cis-MR show promise, optimal SNP selection remains an active area of research without consensus [29, 30]. Therefore, we opted not to implement multi-SNP MR analysis in the present work and recognise that pleiotropy bias cannot be fully excluded and causal interpretations should be made accordingly. Second, instruments based on nonsynonymous variants warrant special caution as structural changes can alter epitope binding. Replication using mass spectrometry-based proteomics may provide future orthogonal validation.

To conclude, we used a genome-wide, proteome-wide approach, agnostic to observational epidemiological associations, to identify proteins that may be potentially causal for lung function. We were able to replicate the vast majority of these and highlight a role for the SNPs instrumenting them in relevant clinical phenotypes. Together with other sources of information to inform efficacy and safety, our findings could inform the choice of therapeutic targets for chronic lung diseases such as COPD.

## Data Availability

UK Biobank data, including data on genomics, proteomics and covariates used in our genome-wide association study (GWAS) analyses, are available to approved *bona fide* researchers *via* application to UK Biobank. Summary statistics for the GWAS of lung function used in these analyses (without UK Biobank included) are publicly available to download from the European Bioinformatics Institute GWAS catalogue at https://www.ebi.ac.uk/gwas/studies/GCST90292613, https://www.ebi.ac.uk/gwas/studies/GCST90292614, https://www.ebi.ac.uk/gwas/studies/GCST90292615 and https://www.ebi.ac.uk/gwas/studies/GCST90292616.

## Supplementary material

**Please note:** supplementary material is not edited by the Editorial Office, and is uploaded as it has been supplied by the author. Supplementary methods 00828-2025.SUPPLEMENT Supplementary tables 00828-2025.SUPPLEMENT Figure S1 00828-2025.SUPPLEMENT2

## References

[1] Vos T, Lim SS, Abbafati C, et al. Global burden of 369 diseases and injuries in 204 countries and territories, 1990–2019: a systematic analysis for the Global Burden of Disease Study 2019. Lancet 2020; 396: 1204–1222. doi:10.1016/S0140-6736(20)30925-9 33069326 PMC7567026

[2] Mullard A. FDA approves first monoclonal antibody for COPD. Nat Rev Drug Discov 2024; 23: 805. doi:10.1038/d41573-024-00164-7 39354151

[3] Sakornsakolpat P, Prokopenko D, Lamontagne M, et al. Genetic landscape of chronic obstructive pulmonary disease identifies heterogeneous cell-type and phenotype associations. Nat Genet 2019; 51: 494–505. doi:10.1038/s41588-018-0342-2 30804561 PMC6546635

[4] Shrine N, Guyatt AL, Erzurumluoglu AM, et al. New genetic signals for lung function highlight pathways and chronic obstructive pulmonary disease associations across multiple ancestries. Nat Genet 2019; 51: 481–493. doi:10.1038/s41588-018-0321-7 30804560 PMC6397078

[5] Shrine N, Izquierdo AG, Chen J, et al. Multi-ancestry genome-wide association analyses improve resolution of genes and pathways influencing lung function and chronic obstructive pulmonary disease risk. Nat Genet 2023; 55: 410–422. doi:10.1038/s41588-023-01314-0 36914875 PMC10011137

[6] Gadd DA, Hillary RF, Kuncheva Z, et al. Blood protein levels predict leading incident diseases and mortality in UK Biobank. medRxiv 2023; 4: 939–948. 2023.05.01.23288879. 10.1038/s43587-024-00655-7 PMC11257969 38987645

[7] Swerdlow DI, Kuchenbaecker KB, Shah S, et al. Selecting instruments for Mendelian randomization in the wake of genome-wide association studies. Int J Epidemiol 2016; 45: 1600–1616. doi:10.1093/ije/dyw088 27342221 PMC5100611

[8] Davey Smith G, Hemani G. Mendelian randomization: genetic anchors for causal inference in epidemiological studies. Hum Mol Genet 2014; 23: R89–R98. doi:10.1093/hmg/ddu328 25064373 PMC4170722

[9] Fauman EB, Hyde C. An optimal variant to gene distance window derived from an empirical definition of cis and trans protein QTLs. BMC Bioinformatics 2022; 23: 169. doi:10.1186/s12859-022-04706-x 35527238 PMC9082853

[10] Aggarwal M, Hwang SJ, Lee DH, et al. Plasma protein biomarkers of spirometry measures of impaired lung function. Chest 2024; 167: 1557–1577. 39580111 10.1016/j.chest.2024.11.012 PMC12202788

[11] Su C-Y, Graaf A, Zhang W, et al. Multi-ancestry proteome-phenome-wide Mendelian randomization offers a comprehensive protein-disease atlas and potential therapeutic targets. medRxiv 2024; preprint [10.1101/2024.10.17.24315553].

[12] Sun BB, Chiou J, Traylor M, et al. Plasma proteomic associations with genetics and health in the UK Biobank. Nature 2023; 622: 329–338. doi:10.1038/s41586-023-06592-6 37794186 PMC10567551

[13] Mbatchou J, Barnard L, Backman J, et al. Computationally efficient whole-genome regression for quantitative and binary traits. Nat Genet 2021; 53: 1097–1103. doi:10.1038/s41588-021-00870-7 34017140

[14] Lawlor DA, Harbord RM, Sterne JA, et al. Mendelian randomization: using genes as instruments for making causal inferences in epidemiology. Stat Med 2008; 27: 1133–1163. doi:10.1002/sim.3034 17886233

[15] Ferkingstad E, Sulem P, Atlason BA, et al. Large-scale integration of the plasma proteome with genetics and disease. Nat Genet 2021; 53: 1712–1721. doi:10.1038/s41588-021-00978-w 34857953

[16] Sun BB, Maranville JC, Peters JE, et al. Genomic atlas of the human plasma proteome. Nature 2018; 558: 73–79. doi:10.1038/s41586-018-0175-2 29875488 PMC6697541

[17] Ishii T, Hagiwara K, Kamio K, et al. Involvement of surfactant protein D in emphysema revealed by genetic association study. Eur J Hum Genet 2012; 20: 230–235. doi:10.1038/ejhg.2011.183 21934714 PMC3260918

[18] Saunders GRB, Wang X, Chen F, et al. Genetic diversity fuels gene discovery for tobacco and alcohol use. Nature 2022; 612: 720–724. doi:10.1038/s41586-022-05477-4 36477530 PMC9771818

[19] Allen RJ, Guillen-Guio B, Oldham JM, et al. Genome-wide association study of susceptibility to idiopathic pulmonary fibrosis. Am J Respir Crit Care Med 2020; 201: 564–574. doi:10.1164/rccm.201905-1017OC 31710517 PMC7047454

[20] Obeidat M, Li X, Burgess S, et al. Surfactant protein D is a causal risk factor for COPD: results of Mendelian randomisation. Eur Respir J 2017; 50: 1700657. doi:10.1183/13993003.00657-2017 29191953 PMC7614619

[21] Salvioni L, Testa F, Sulejmani A, et al. Surfactant protein D (SP-D) as a biomarker of SARS-CoV-2 infection. Clin Chim Acta 2022; 537: 140–145. doi:10.1016/j.cca.2022.10.013 36341812 PMC9617654

[22] COVID-19 Host Genetics Initiative. A first update on mapping the human genetic architecture of COVID-19. Nature 2022; 608: E1–e10. 35922517 10.1038/s41586-022-04826-7 PMC9352569

[23] Jansen R, Hottenga JJ, Nivard MG, et al. Conditional eQTL analysis reveals allelic heterogeneity of gene expression. Hum Mol Genet 2017; 26: 1444–1451. doi:10.1093/hmg/ddx043 28165122 PMC6075455

[24] Raghu G, Mouded M, Prasse A, et al. Randomized phase IIa clinical study of an anti-α(v)β(6) monoclonal antibody in idiopathic pulmonary fibrosis. Am J Respir Crit Care Med 2022; 206: 1166–1168. doi:10.1164/rccm.202205-0868LE 35830489 PMC9704833

[25] Rathore N, Ramani SR, Pantua H, et al. Paired immunoglobulin-like Type 2 Receptor Alpha G78R variant alters ligand binding and confers protection to Alzheimer's disease. PLoS Genet 2018; 14: e1007427. doi:10.1371/journal.pgen.1007427 30388101 PMC6235402

[26] Pietzner M, Wheeler E, Carrasco-Zanini J, et al. Mapping the proteo-genomic convergence of human diseases. Science 2021; 374: eabj1541. doi:10.1126/science.abj1541 34648354 PMC9904207

[27] Allen RJ, Oldham JM, Jenkins DA, et al. Longitudinal lung function and gas transfer in individuals with idiopathic pulmonary fibrosis: a genome-wide association study. Lancet Respir Med 2023; 11: 65–73. doi:10.1016/S2213-2600(22)00251-X 35985358 PMC10077113

[28] Zheng J, Baird D, Borges MC, et al. Recent developments in Mendelian randomization studies. Curr Epidemiol Rep 2017; 4: 330–345. doi:10.1007/s40471-017-0128-6 29226067 PMC5711966

[29] Lin Z, Pan W. A robust cis-Mendelian randomization method with application to drug target discovery. Nat Commun 2024; 15: 6072. doi:10.1038/s41467-024-50385-y 39025905 PMC11258283

[30] Gkatzionis A, Burgess S, Newcombe PJ. Statistical methods for cis-Mendelian randomization with two-sample summary-level data. Genet Epidemiol 2023; 47: 3–25. doi:10.1002/gepi.22506 36273411 PMC7614127

