# Supplementary material for: Proteome-wide Mendelian randomisation of lung function to identify potential therapeutic targets for respiratory disease
Source: ERJ Open Res. 2026 Sep 1;12(5):00828-2025. doi: 10.1183/23120541.00828-2025 (PMC13531259; doi:10.1183/23120541.00828-2025)
Supplement: Supplementary file 1 [file 00828-2025.SUPPLEMENT.pdf]

Supplementary Methods

Colocalization analysis

The prioritized MR results were evaluated by Bayesian colocalization.[1] For genomic regions with multiple signals, we performed conditional analyses using GCTA-COJO[2] using an LD reference panel of 10,000 randomly selected UK Biobank European ancestry participants. We prioritised MR findings for which the posterior probability (PP) of a shared signal was >0.7[3] and where visual inspection of the mirror ("Miami") plot was consistent with colocalization of the pQTL and lung function GWAS signal (**Supplementary Figure 1** and **Supplementary Table 1**).

Reverse causation test of single-cis-MR findings

To explore possible reverse causation, i.e. whether a protein level could be causally influenced by the lung function trait under study, we performed MR in the opposite direction.[4] For each protein identified in the protein→lung function MR analysis, we chose the lung function trait for which there was strongest evidence of causality by that protein. We identified independent lung function signals for the relevant trait from Shrine et al.[5] (independent SNPs associated at $P<5x10^{-9}$), and applied Steiger filtering[6] to remove SNPs that explained more variance in the protein levels than in the lung function trait. We used the remaining SNPs as instruments for the lung function trait, and used the inverse variance-weighted (IVW) MR method (as implemented by mr_ivw() in the Two-Sample MR package) to estimate the causal effect of that lung function trait on the relevant protein.

Respiratory-wide association study

To determine the clinical relevance of the causal associations identified with lung function, we performed a respiratory phenome-wide association study (PheWAS), querying each cis-pQTL against 28 GWASs (**Supplementary Table 2**) for: asthma, bronchiectasis, bronchopneumonia, chronic bronchitis, COPD, chronic sputum production, emphysema, idiopathic pulmonary fibrosis (IPF), respiratory infections, interstitial lung abnormalities (ILA) and pneumothorax. We report associations with *p*-value <0.01 (FDR adjusted *p*-values are provided in **Supplementary Table 5**).

PheWAS

For proteins identified in the single-cis MR analysis, we undertook single-variant PheWAS for the cis-pQTLs selected as instruments across 1,909 traits in up to 430,402 unrelated EUR individuals in the UK Biobank using Deep-PheWAS (PMID 36744935). The PheWAS results were aligned to protein-increasing alleles. We report associations with FDR<1%.

1. Giambartolomei, C. *et al.* Bayesian test for colocalisation between pairs of genetic association studies using summary statistics. *PLoS Genet* **10**, e1004383 (2014).
2. Yang, J. *et al.* Conditional and joint multiple-SNP analysis of GWAS summary statistics identifies additional variants influencing complex traits. *Nat Genet* **44**, 369-75, s1-3 (2012).
3. Zhang, L.Y. *et al.* Systematic Druggable Genome-Wide Mendelian Randomization Identifies Therapeutic Targets for Functional Outcome After Ischemic Stroke. *Journal of the American Heart Association* **13**, e034749 (2024).
4. Timpson, N.J. *et al.* C-reactive protein levels and body mass index: elucidating direction of causation through reciprocal Mendelian randomization. *Int J Obes (Lond)* **35**, 300-8 (2011).
5. Shrine, N. *et al.* Multi-ancestry genome-wide association analyses improve resolution of genes and pathways influencing lung function and chronic obstructive pulmonary disease risk. *Nat Genet* **55**, 410-422 (2023).
6. Hemani, G., Tilling, K. & Davey Smith, G. Orienting the causal relationship between imprecisely measured traits using GWAS summary data. *PLoS Genet* **13**, e1007081 (2017).
