# Supplementary material for: Proteome-wide Mendelian randomisation of lung function to identify potential therapeutic targets for respiratory disease
Source: ERJ Open Res. 2026 Sep 1;12(5):00828-2025. doi: 10.1183/23120541.00828-2025 (PMC13531259; doi:10.1183/23120541.00828-2025)

Supplementary Figure 1

Mirror plots to compare the region plots of protein-genetic associations with those of lung function-genetic associations for visualization of colocalization. Each point is a genetic variant. Points above the x axis show the strength of association with the lung function trait and points below the x axis show the strength of association with the protein level. Variants are coloured by their linkage disequilibrium with the cis-pQTL.

EFEMP1 rs59985551

EFEMP1_FEV1_2_56106928_C_T

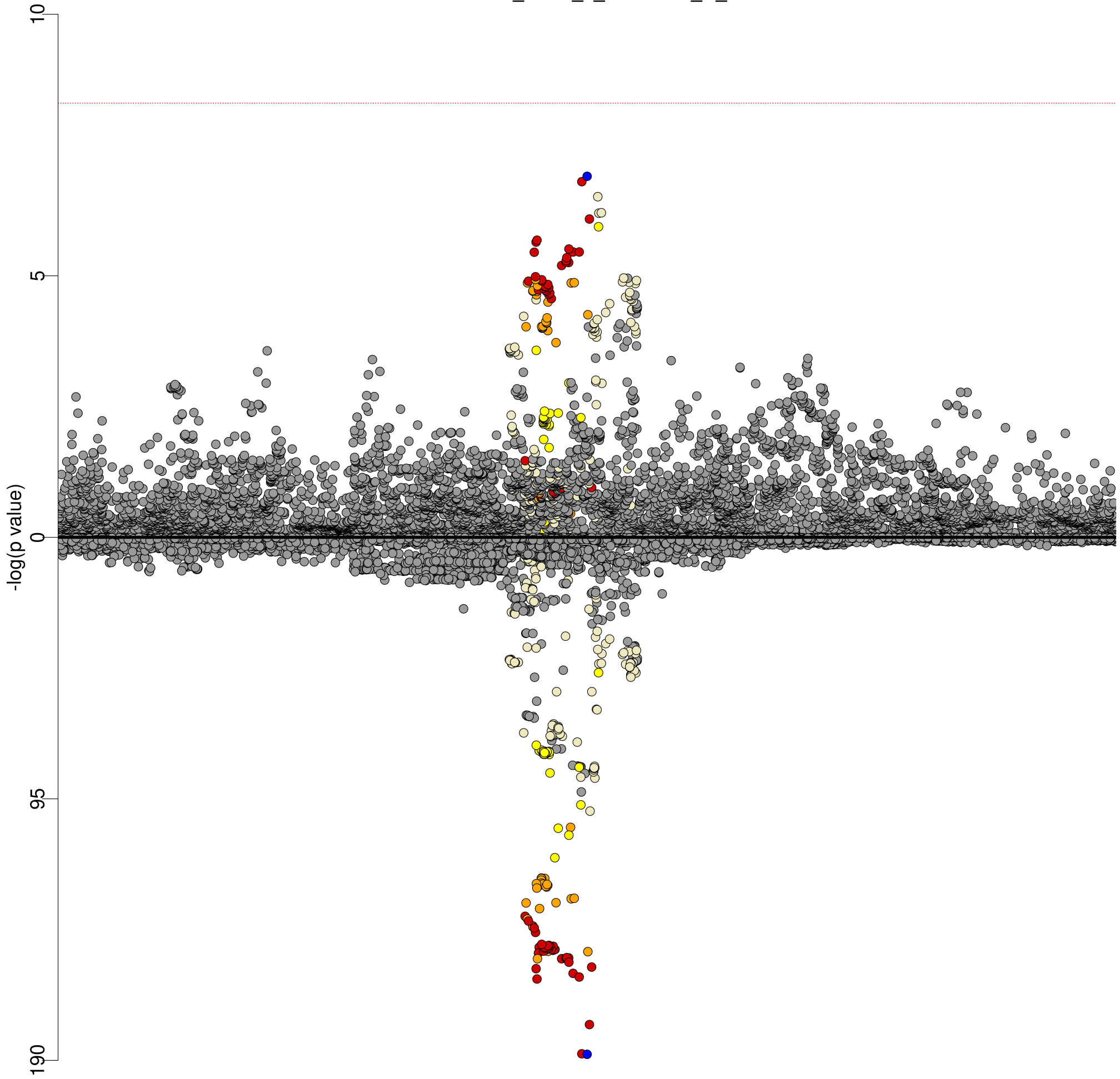

EFEMP1_FVC_2_56106928_C_T

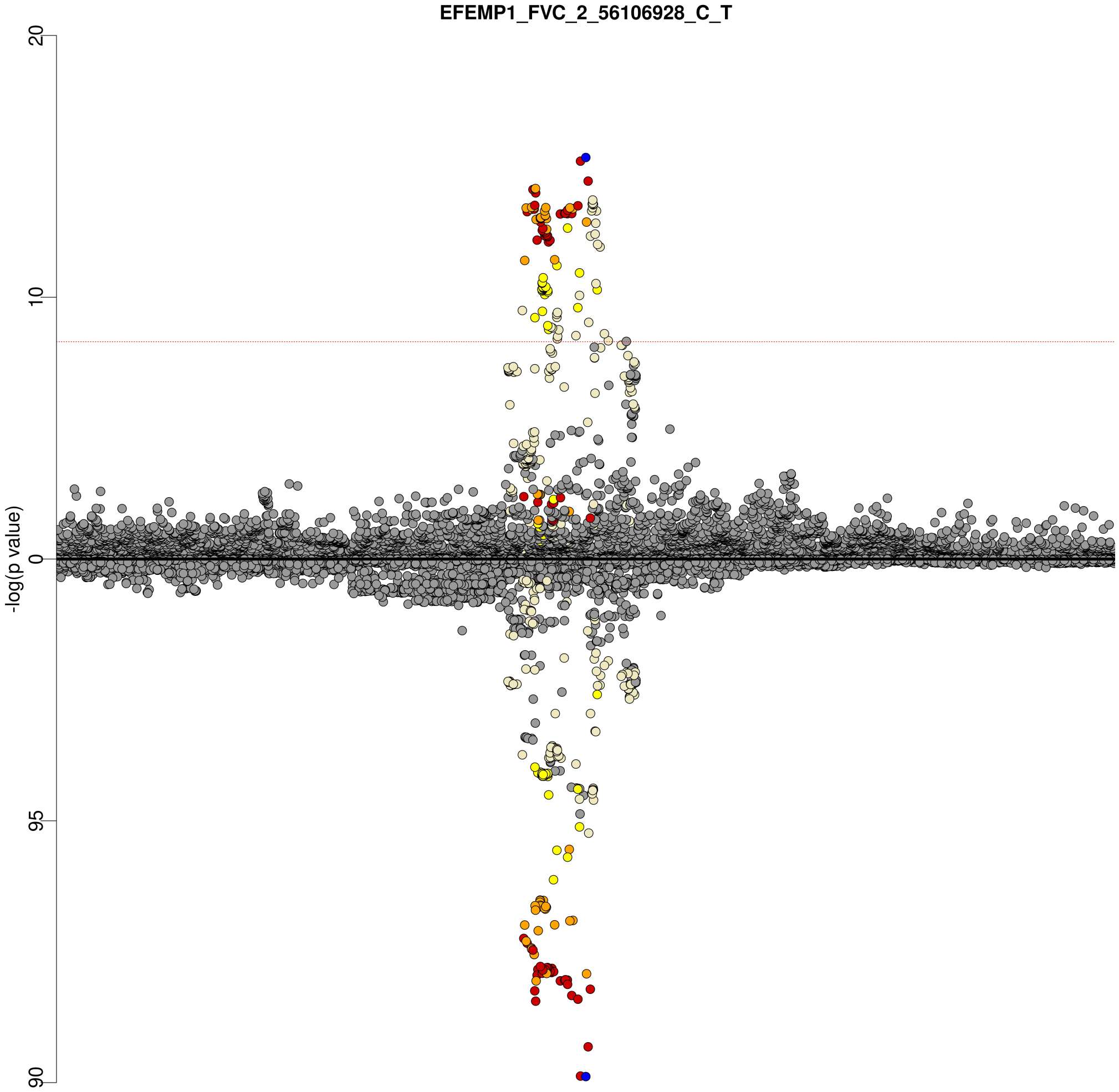

COL6A3 rs1050785

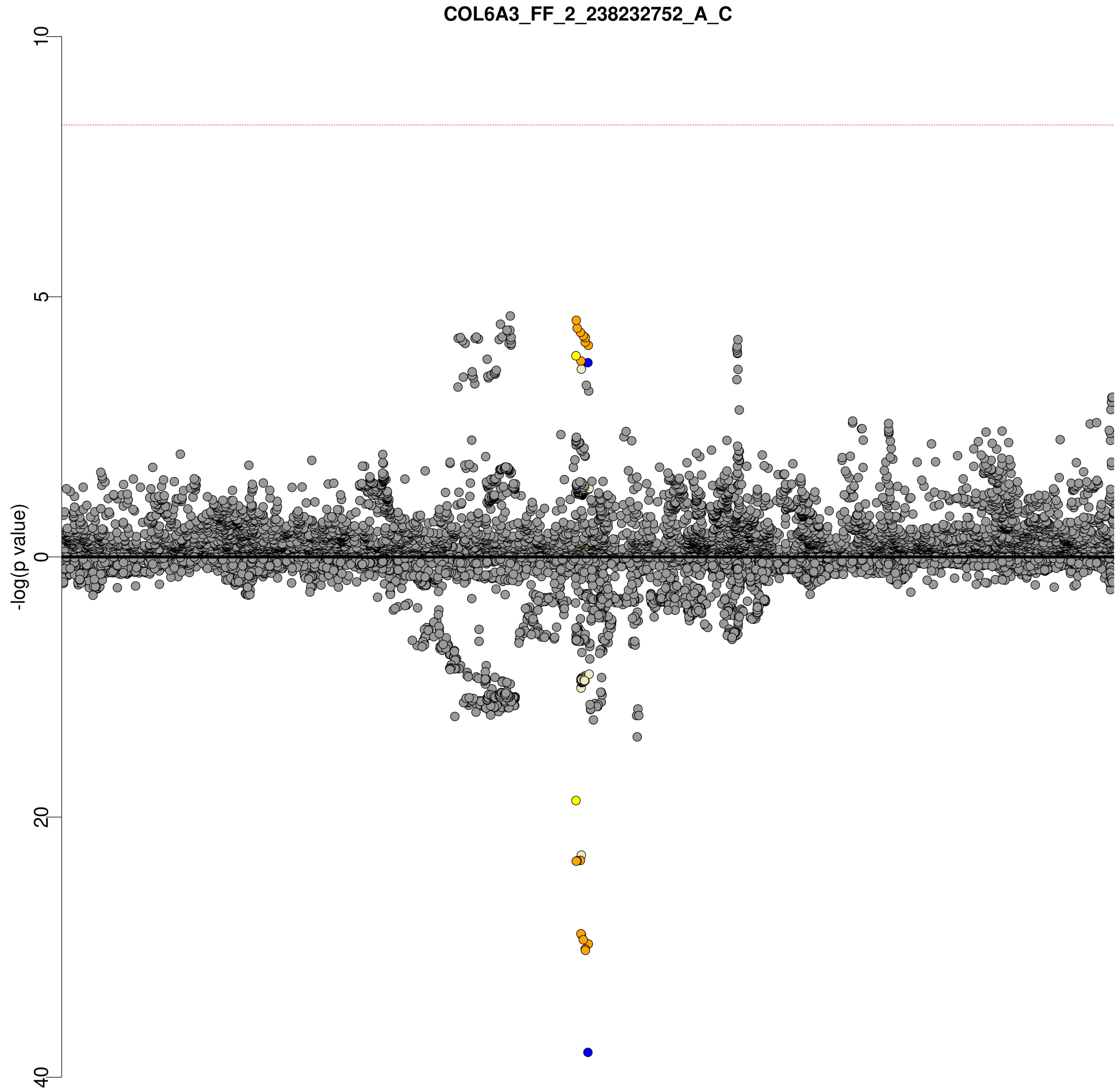

ITGAV rs9333290

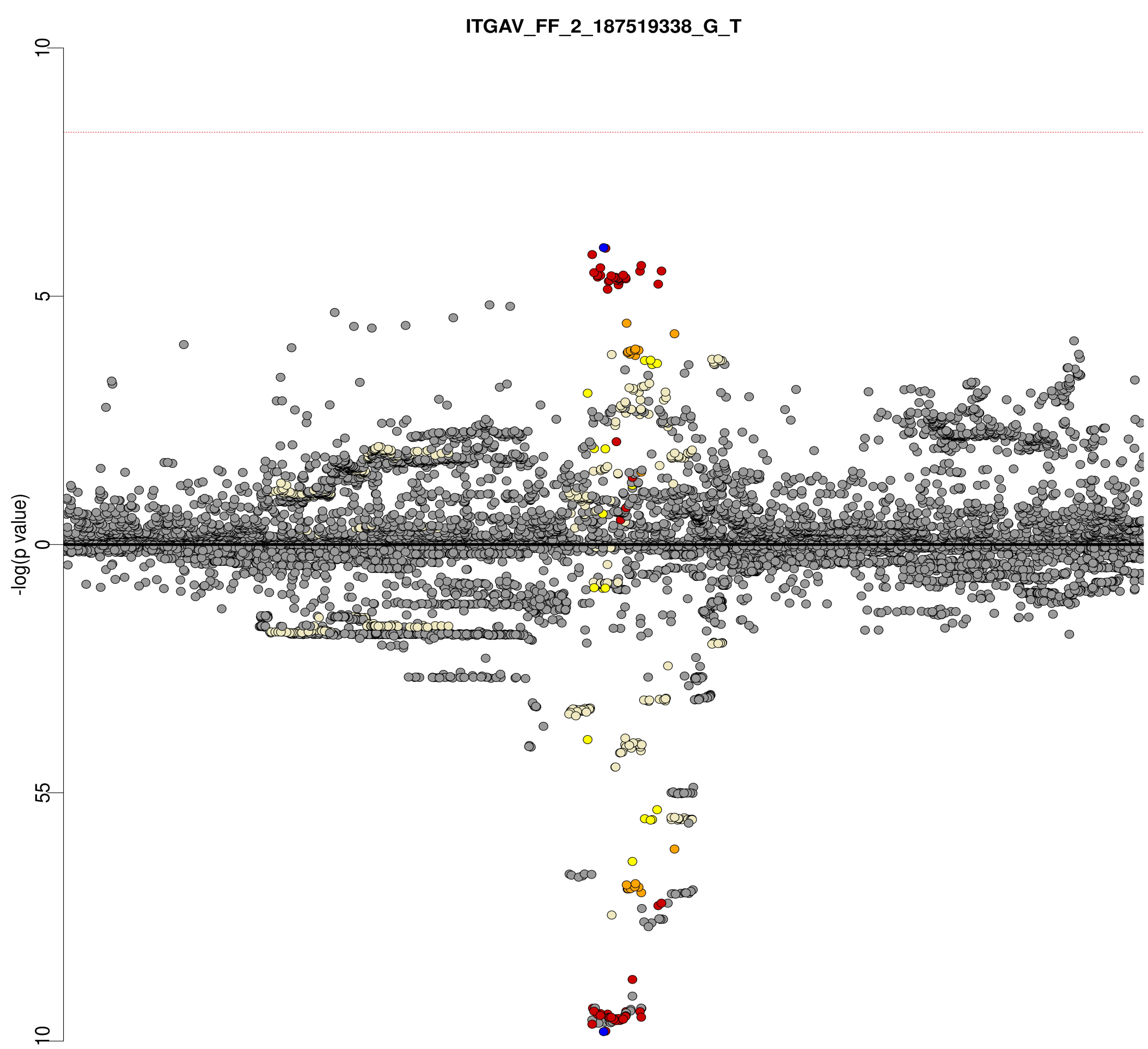

TNFRSF6B rs6062497

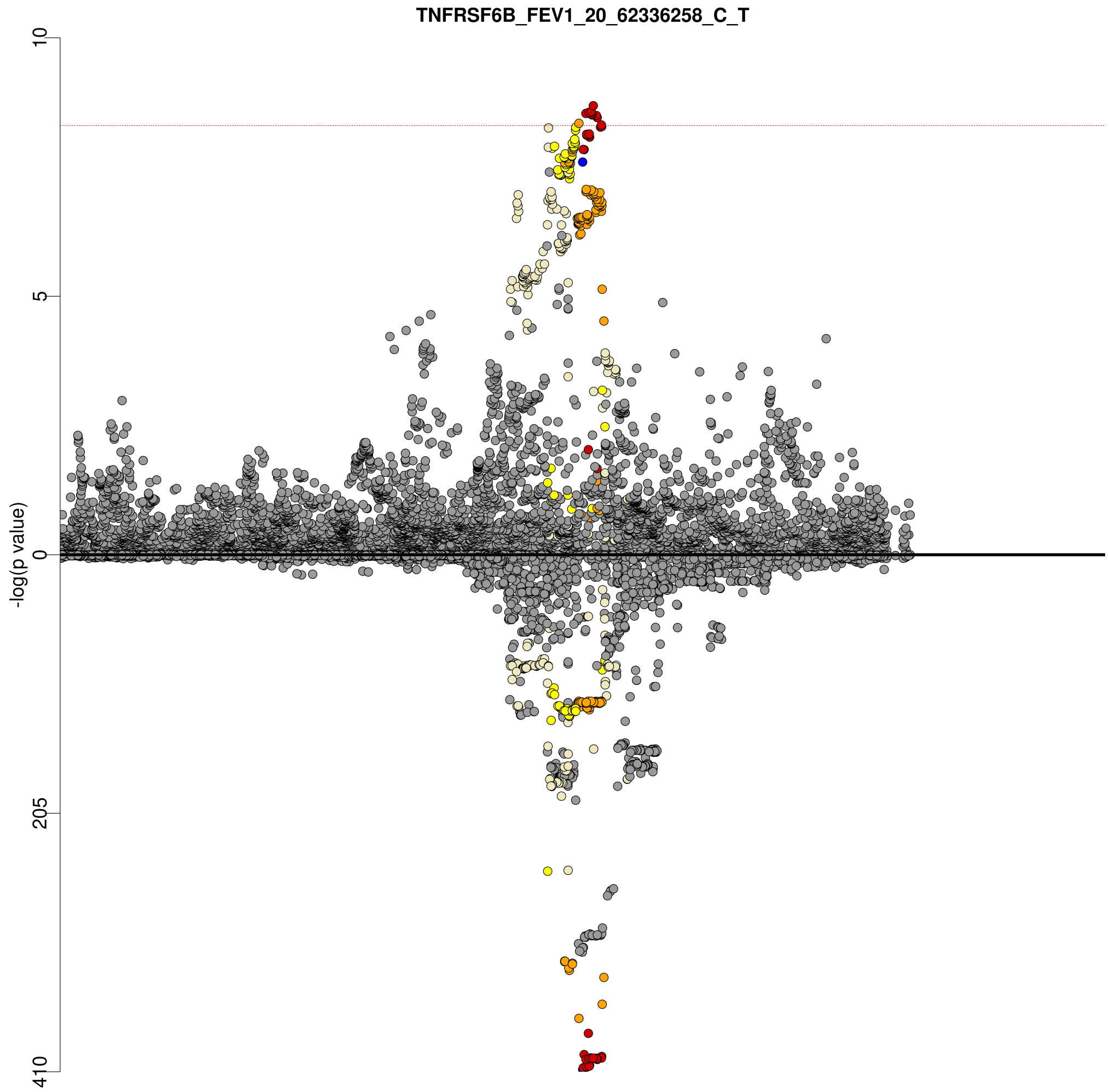

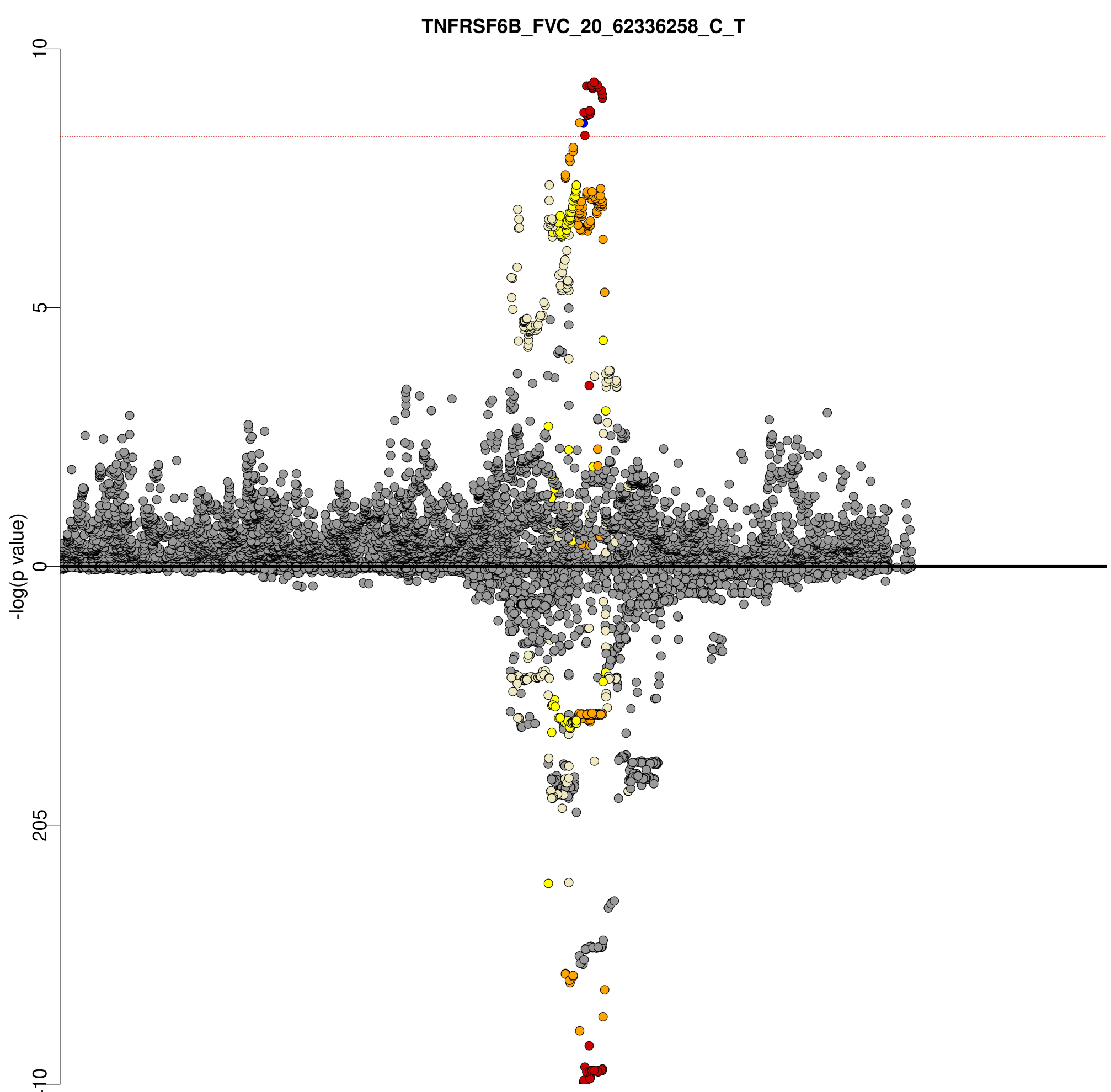

NUDT5 rs12569964

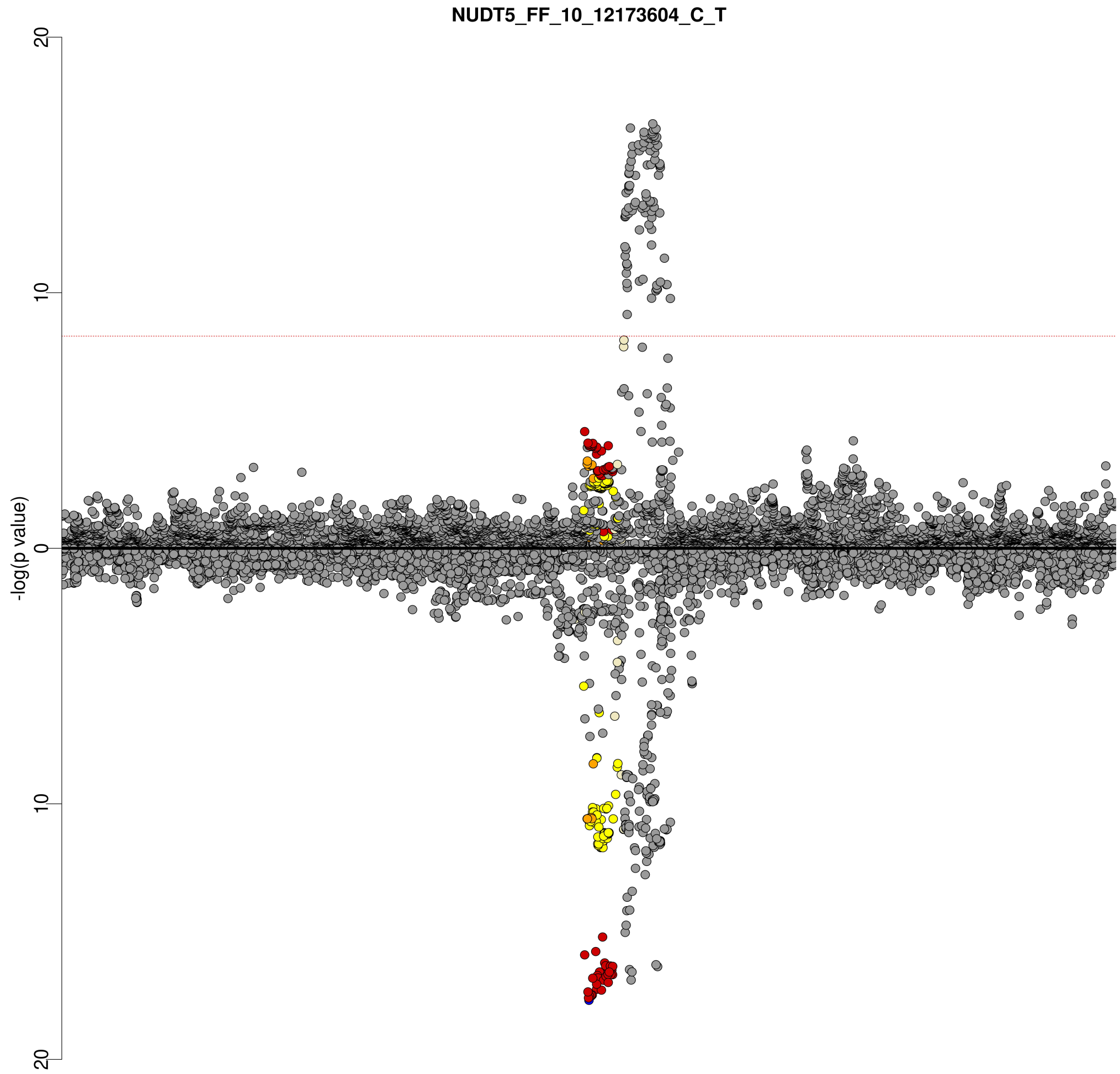

SFTPD rs721917

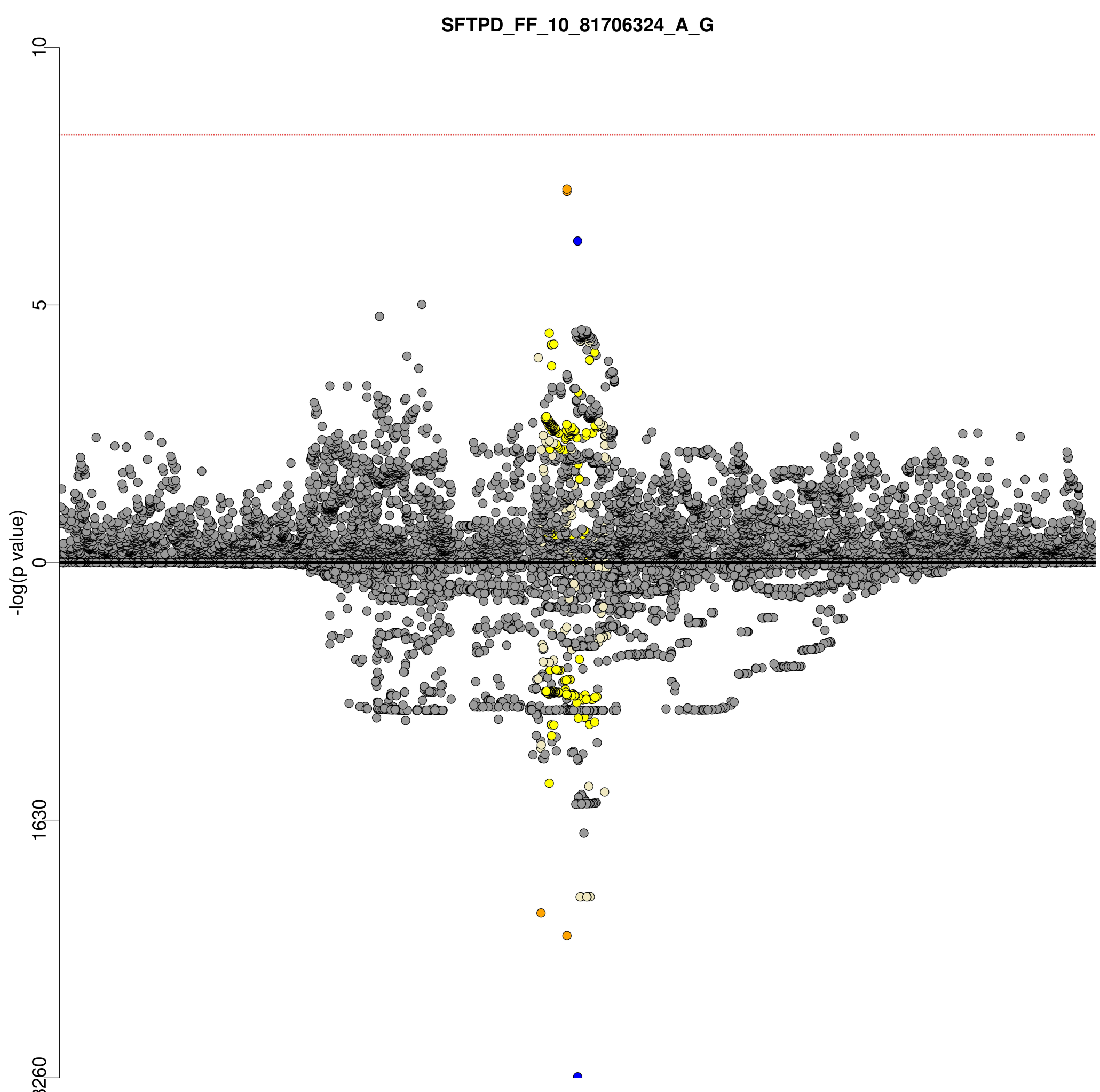

PILRA rs1859788

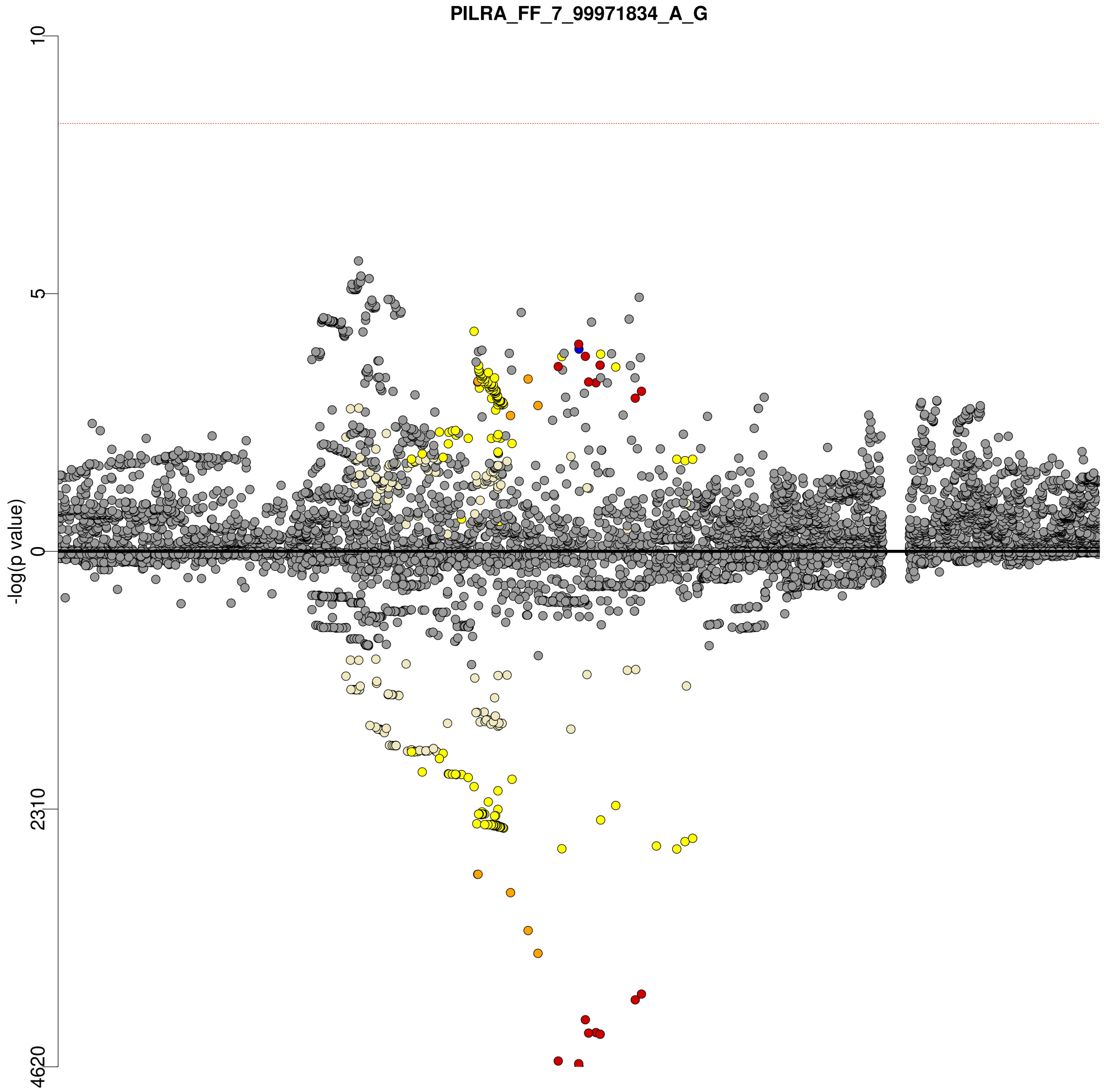

PILRB rs1859788

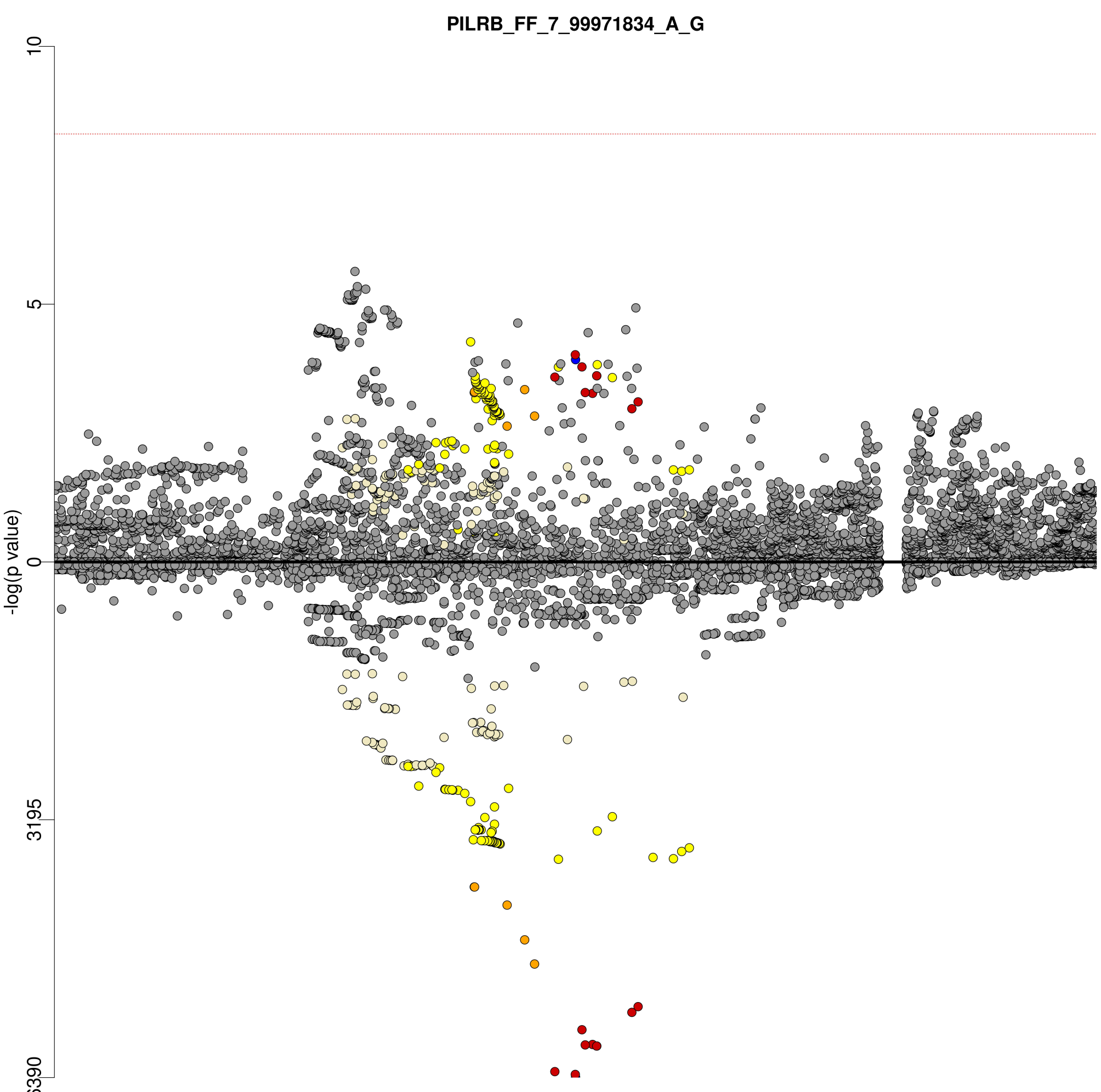

TDRKH rs4845556

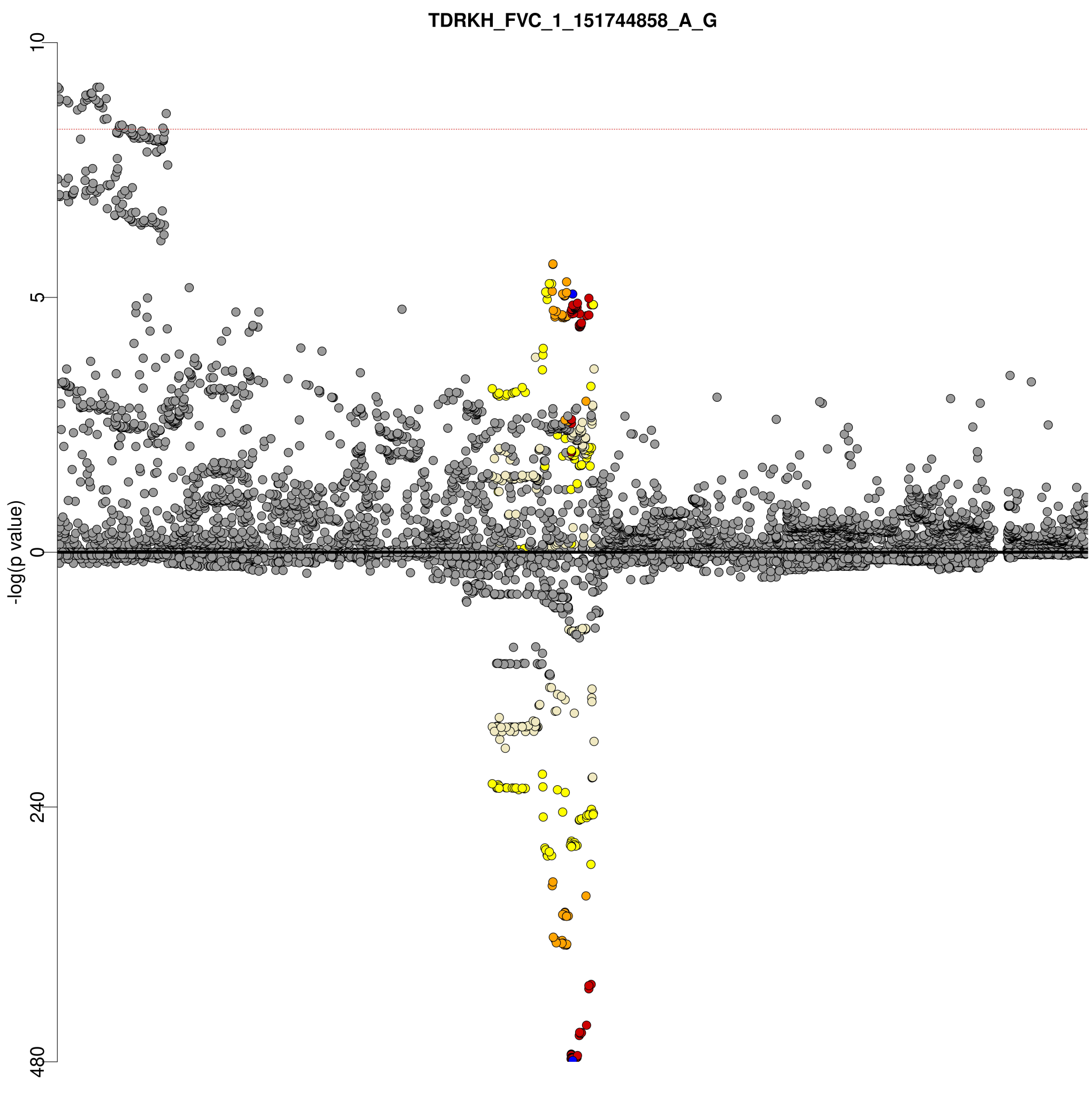

SLMAP rs9814503

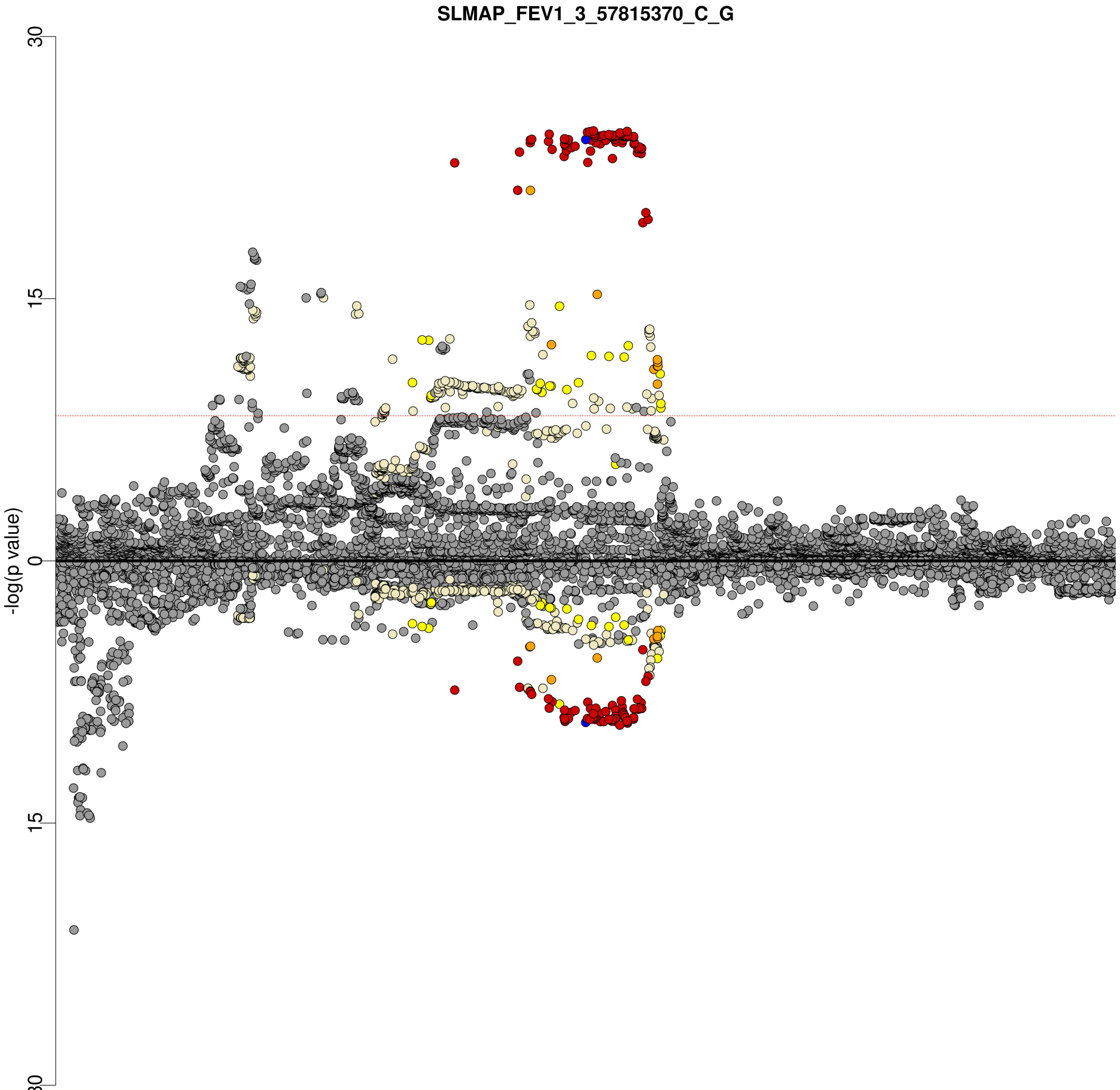

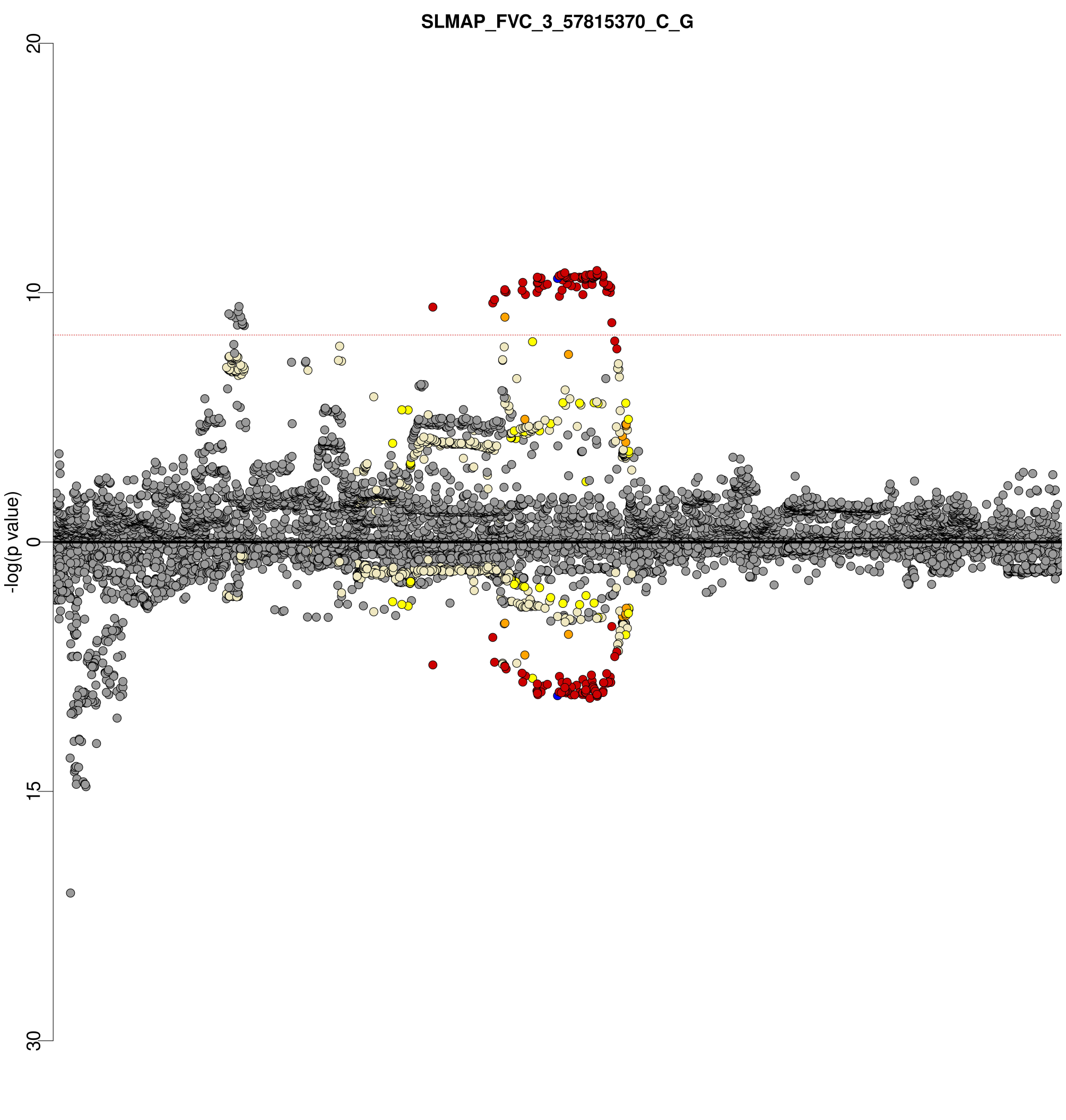

WASHC3 rs11111164

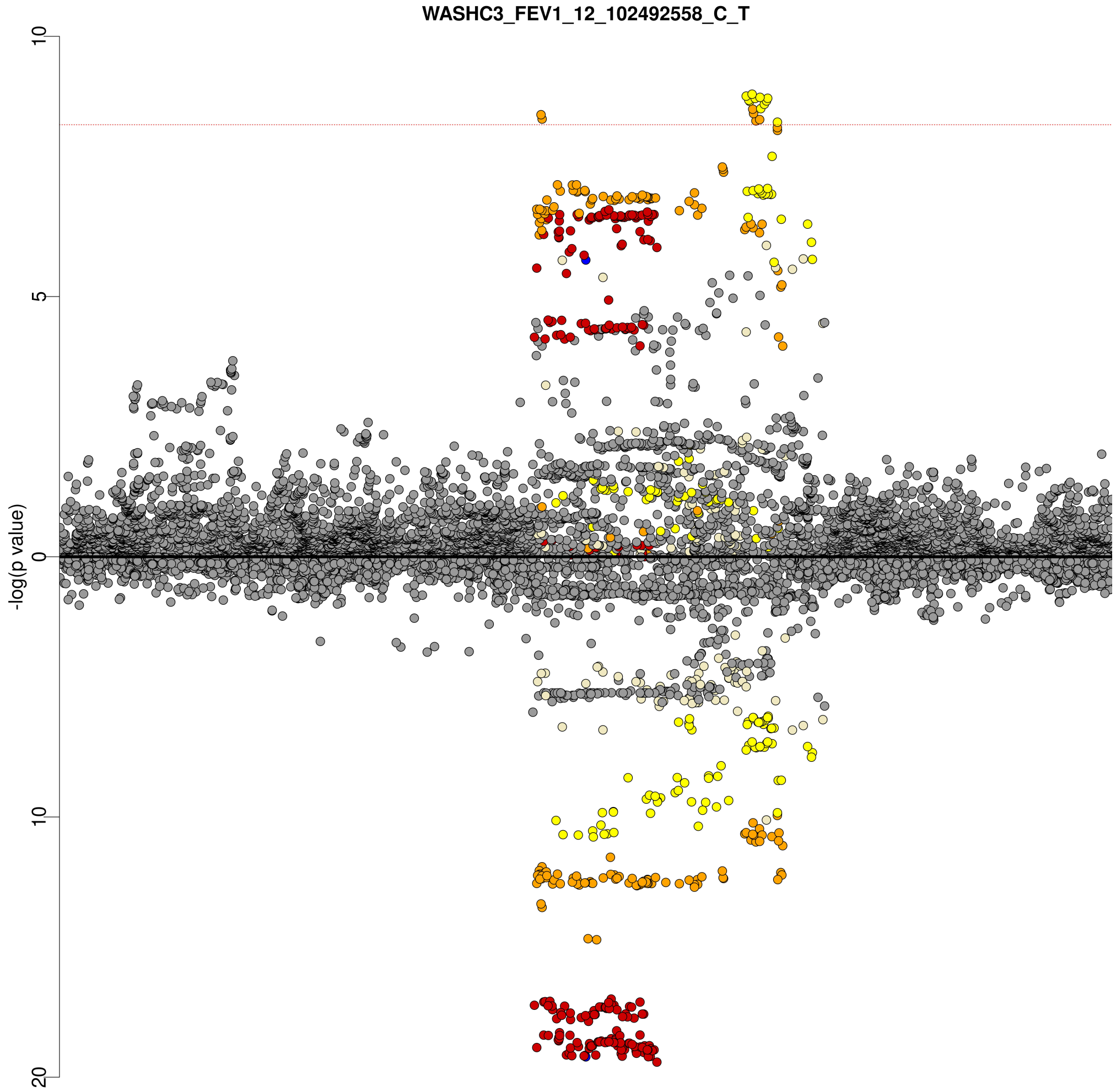

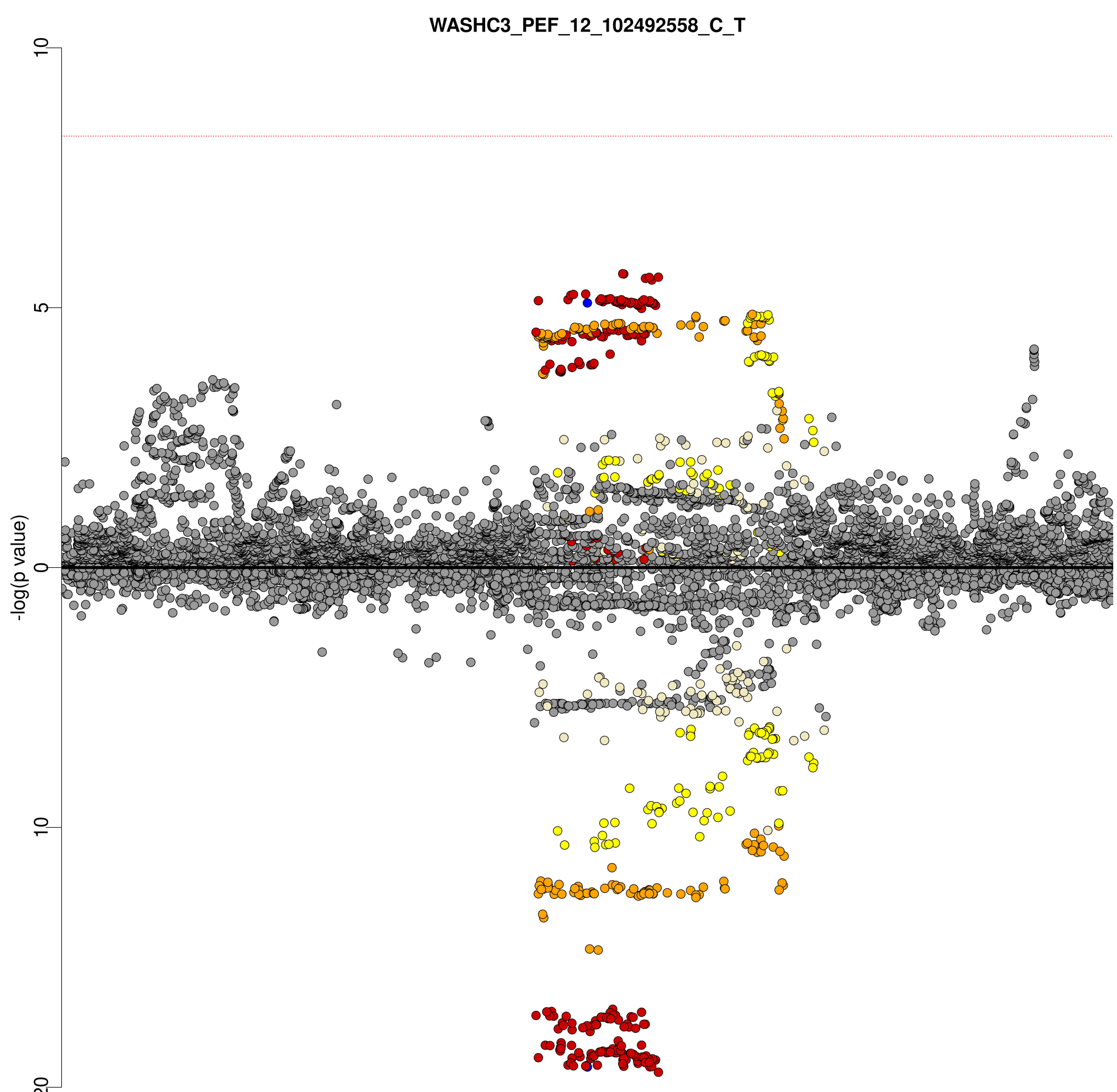

PRSS53 rs7199949

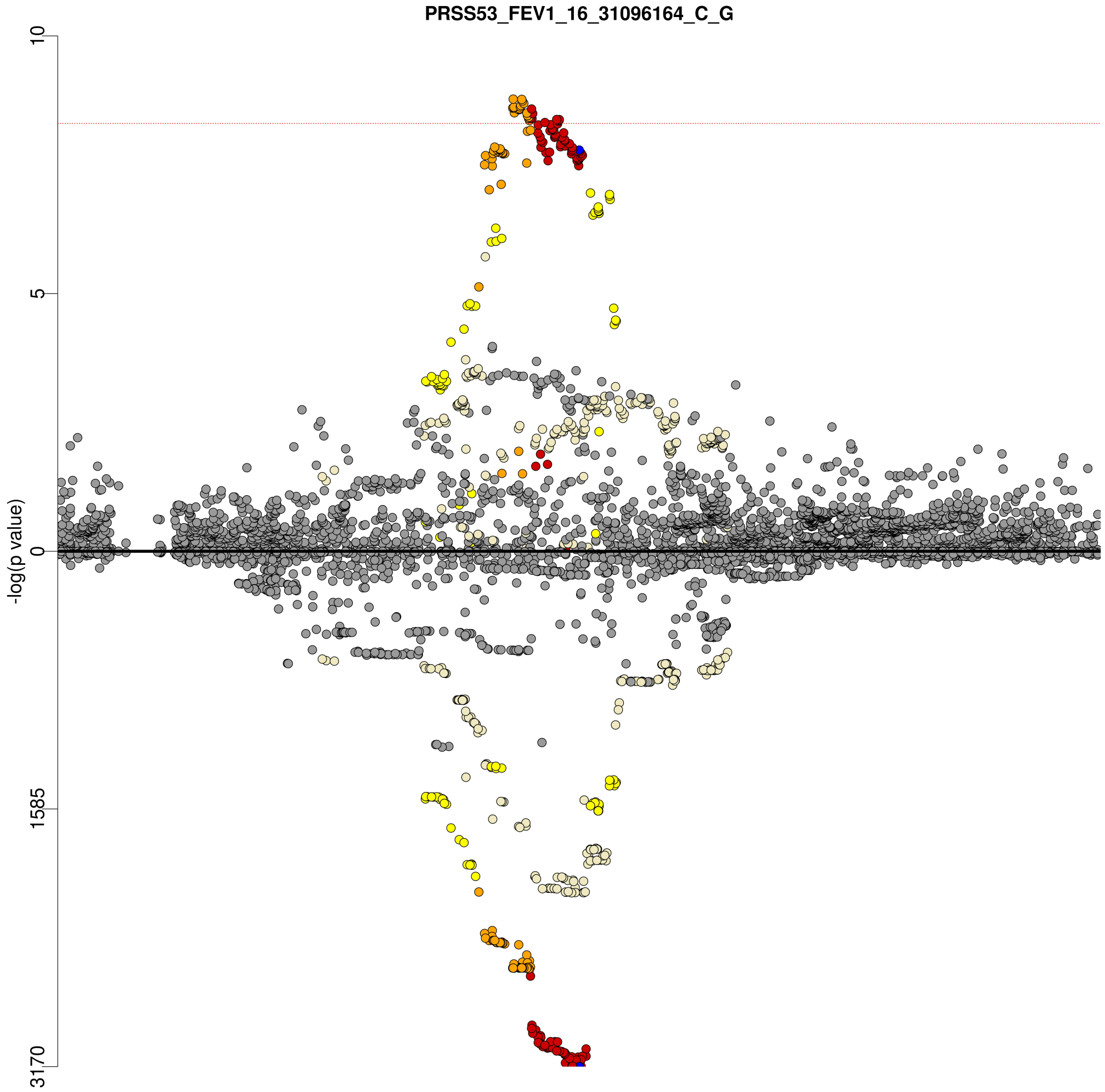

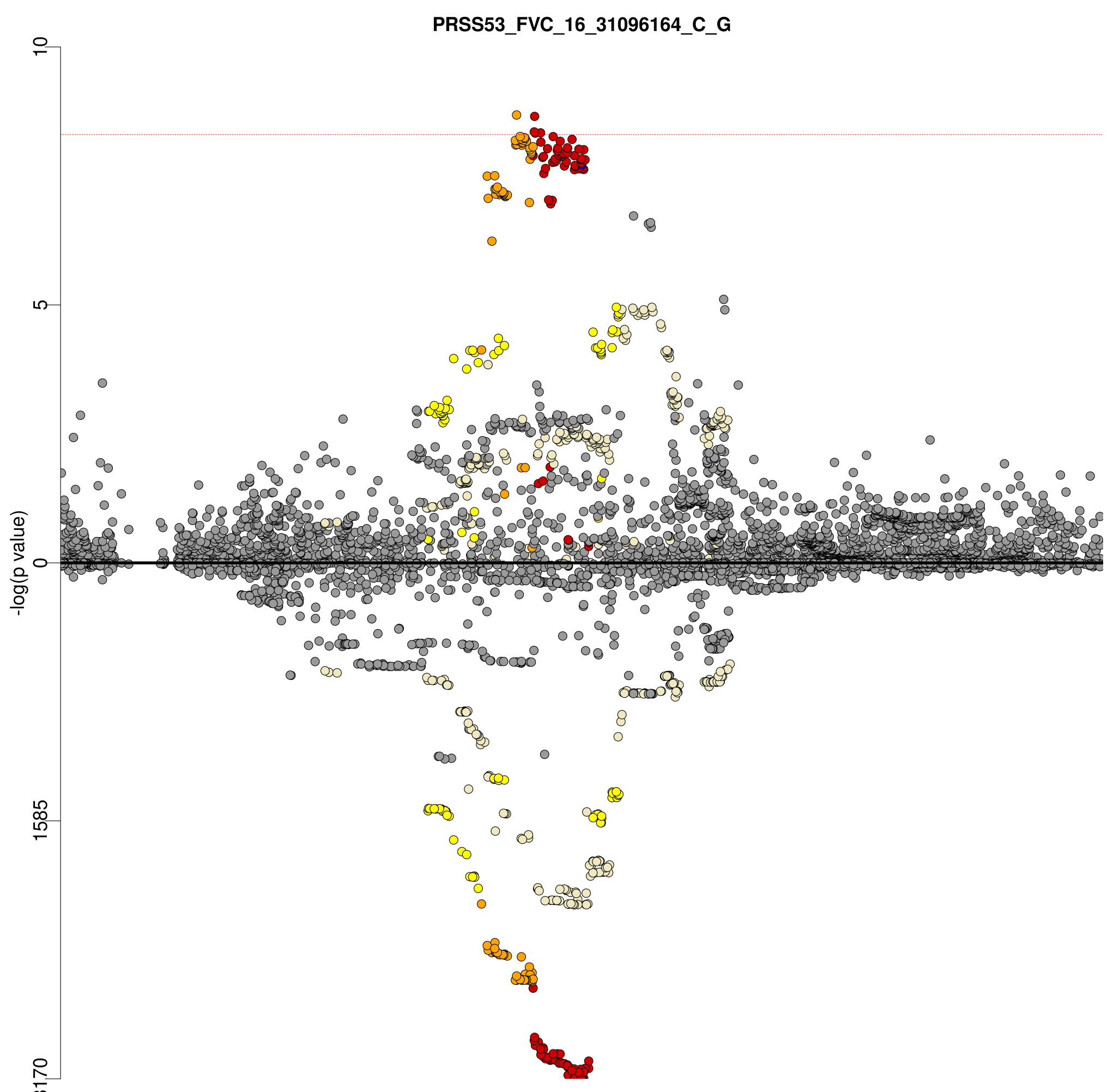

TOP2B rs3806631

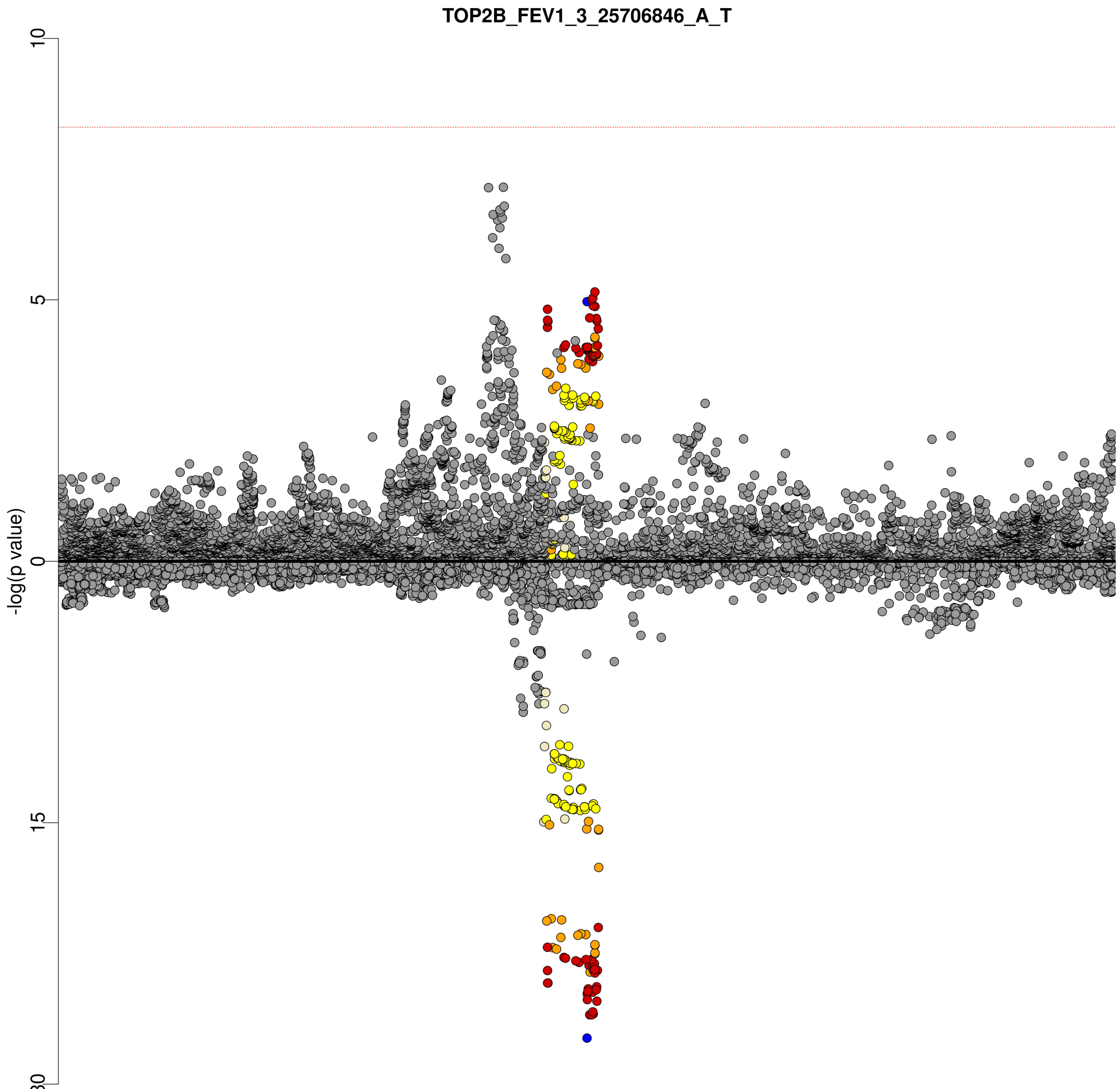

CCND2 rs76895963

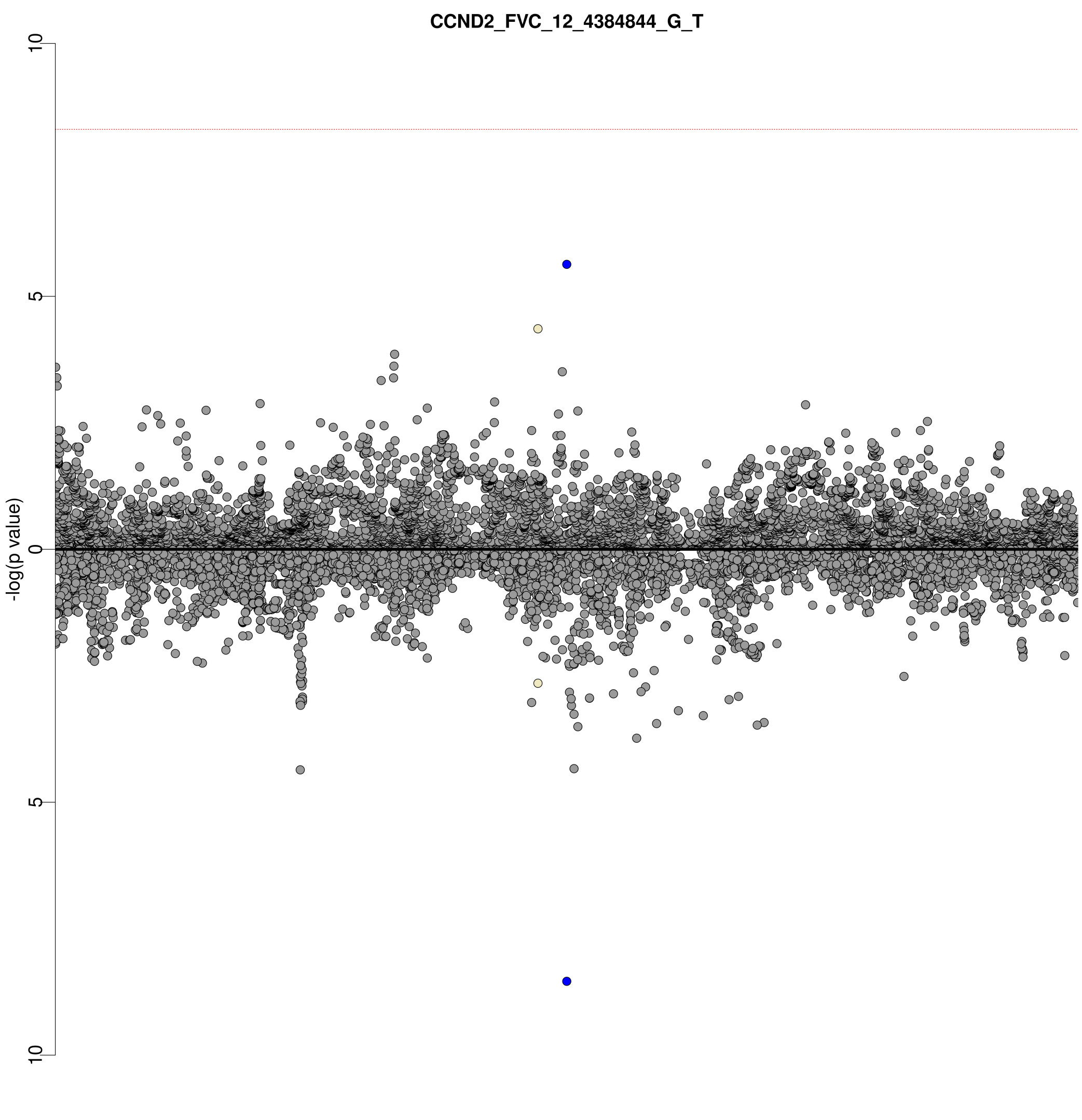

PTPRK rs9375546

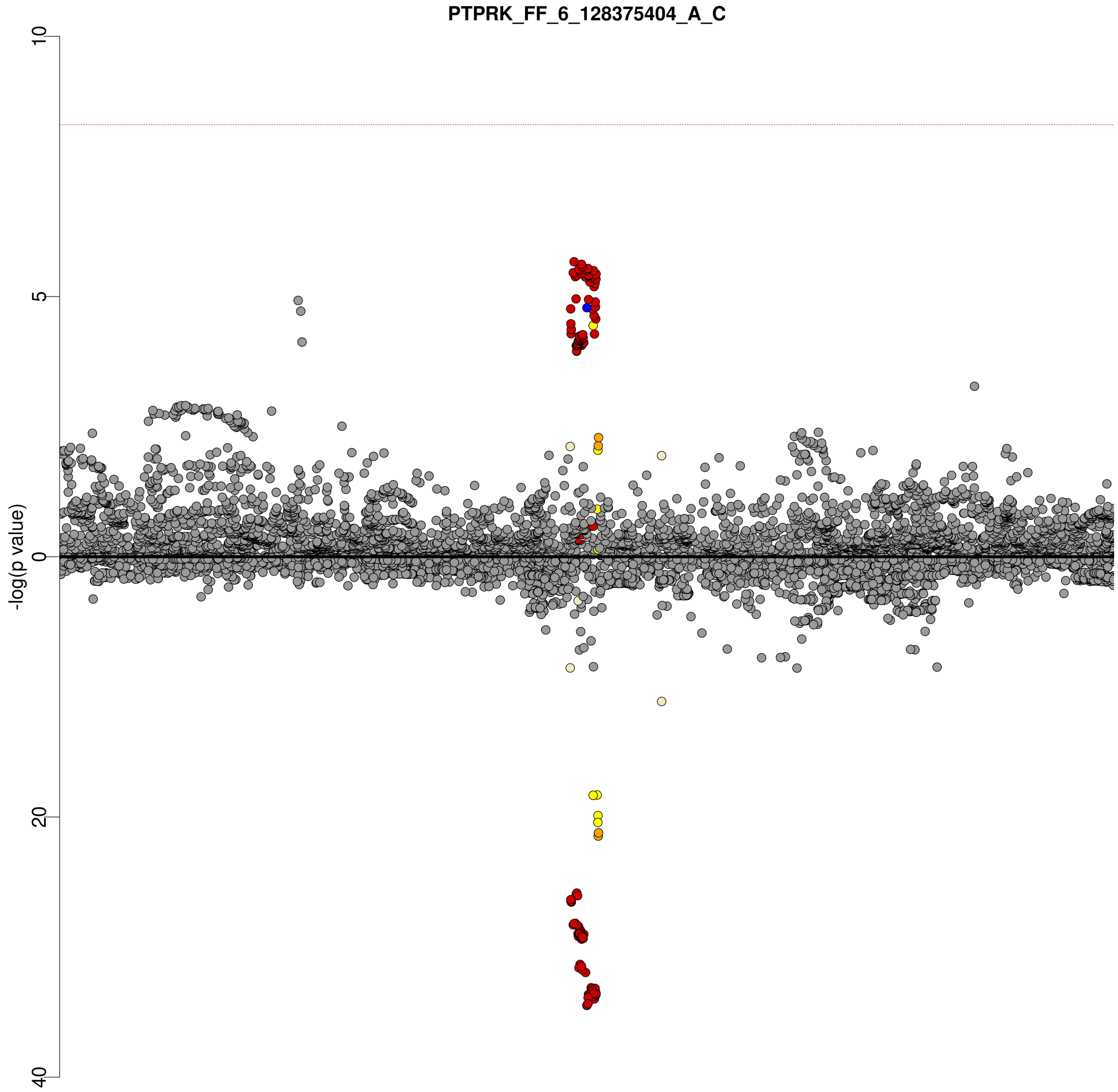

GRHPR rs4878690

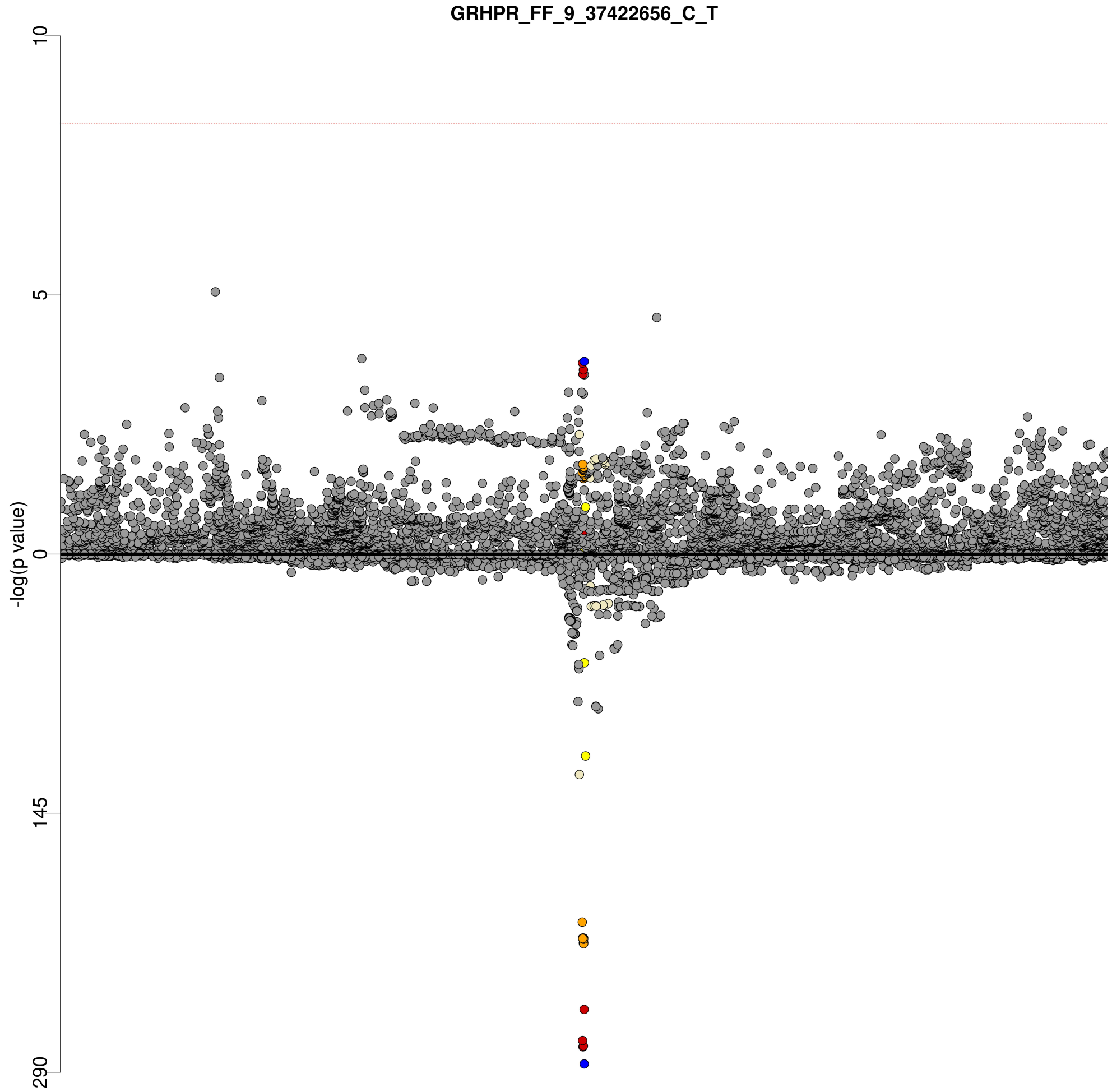

DTD1 rs6081238

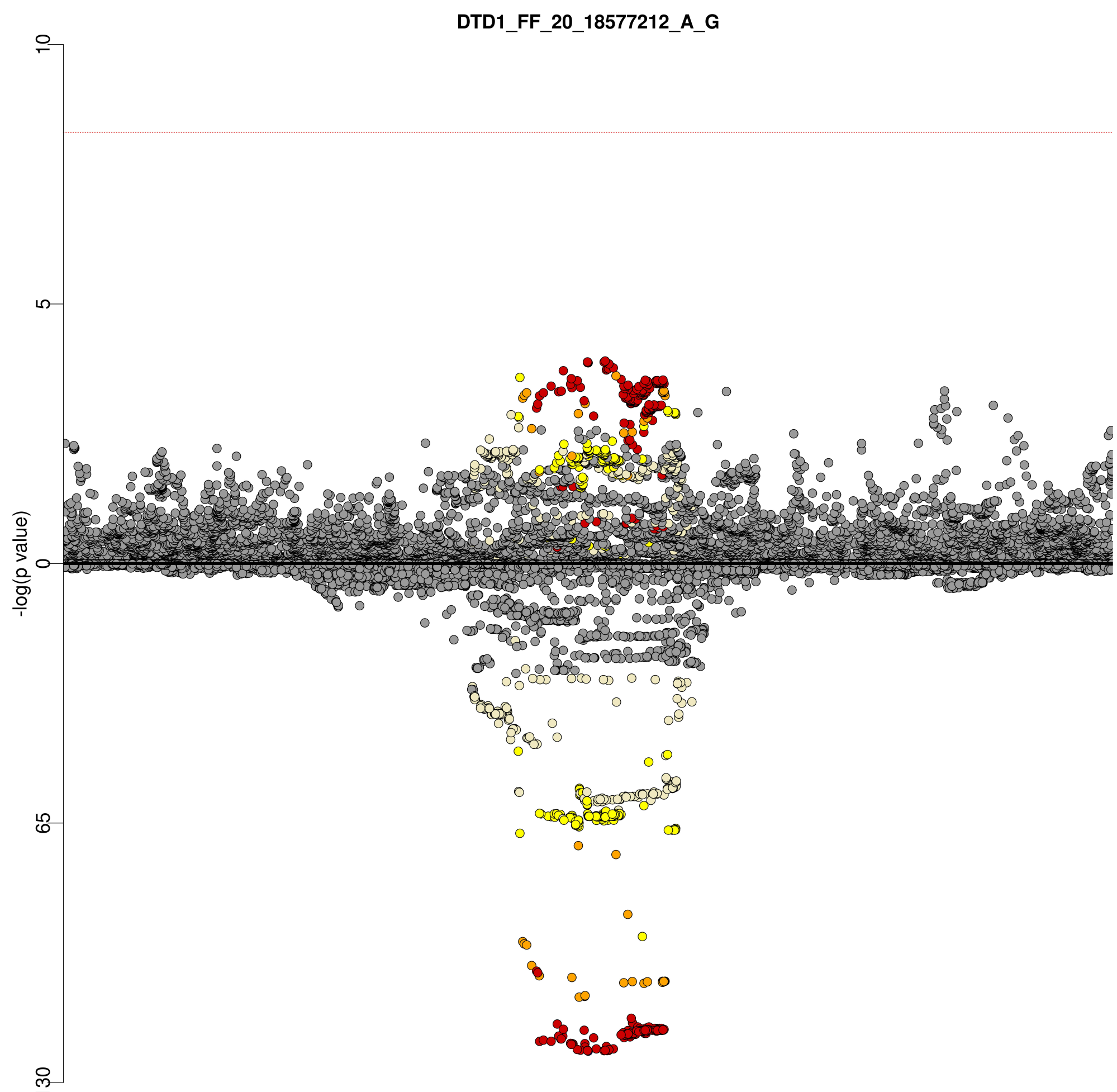

Supplement: Supplementary file 2 [file 00828-2025.SUPPLEMENT2.pdf]
